# Transgressive resistance to *Heterodera glycines* in chromosome segment substitution lines derived from susceptible soybean parents

**DOI:** 10.1002/tpg2.20091

**Published:** 2021-04-05

**Authors:** Minghui Huang, Ruifeng Qin, Chunjie Li, Chunyan Liu, Ye Jiang, Jinyao Yu, Doudou Chang, Philip A. Roberts, Qingshan Chen, Congli Wang

**Affiliations:** ^1^ Key Laboratory of Soybean Molecular Design Breeding Northeast Institute of Geography and Agroecology Chinese Academy of Sciences Harbin Heilongjiang 150081 China; ^2^ University of Chinese Academy of Sciences Beijing 100049 China; ^3^ College of Agronomy Northeast Agricultural University Harbin Heilongjiang 150030 China; ^4^ Department of Nematology University of California Riverside CA 92521 USA

## Abstract

Chromosome segment substitution lines (CSSLs) are valuable genetic resources for quantitative trait loci (QTL) mapping of complex agronomic traits especially suitable for minor effect QTL. Here, 162 BC_3_F_7_–BC_7_F_3_ CSSLs derived from crossing two susceptible parent lines, soybean [*Glycine max* (L.) Merr.] ‘Suinong14’ (recurrent parent) × wild soybean (*G. soja* Siebold & Zucc.) ZYD00006, were used for QTL mapping of soybean cyst nematode (SCN, *Heterodera glycine* Ichinohe) resistance based on female index (FI) and cysts per gram root (CGR) through phenotypic screening and whole‐genome resequencing of CSSLs. Phenotypic results displayed a wide range of distribution and transgressive lines in both HG Type 2.5.7 FI and CGR and demonstrated a higher correlation between CGR and root weight (*R^2^* = .5424) compared with than between FI and CGR (*R^2^* = .0018). Using the single‐marker analysis nonparametric mapping test, 33 significant QTL were detected on 18 chromosomes contributing resistance to FI and CGR. Fourteen QTL contributing 5.6–15.5% phenotypic variance (PVE) to FI were revealed on 11 chromosomes, and 16 QTL accounting for 6.1–36.2% PVE in CGR were detected on 14 chromosomes with strong additive effect by multiple‐QTL model (MQM) mapping. Twenty‐five and 13 out of all 38 QTL identified for FI and CGR on 20 chromosomes were from ZYD00006 and Suinong14, respectively. The CSSLs with the combination of positive alleles for FI, CGR, and root weight exhibited low nematode reproduction. For the first time, QTL associated with CGR have been detected, and both FI and CGR should be considered for breeding purposes in the absence of strong resistance genes such as *rhg1* and *Rhg4*.

## Core Ideas

- CSSLs are suitable for QTL mapping *Heterodera glycines* resistance trait in soybean.
- Transgressive resistance was found in CSSLs.
- For the first time, QTL associated with cysts per gram root have been detected.
- Positive alleles for female index and cysts per gram root exhibited low nematode reproduction.

**Abbreviations**
CGR: cysts per gram root
Chr: chromosome
CSSL: chromosome segment substitution line
FI: female index
GWAS: genome‐wide association study
HG or Hg: *Heterodera glycine*
J2: second‐stage juvenile
LOD: logarithm of odds
MQM: multiple‐QTL model
PVE: phenotypic variance
QTL: quantitative trait loci
RIL: recombinant inbred line
RKN: root‐knot nematode
SCN: soybean cyst nematode
SNP: single nucleoid polymorphism.

## 1 INTRODUCTION

Soybean (*Glycine max* L. Merr.) is one of the most important oil and economic crops worldwide, providing almost 56% of global oil seed as well as rich protein and other nutrition for people and livestock (Wilson, 2008). Soybean cyst nematode (SCN, *Heterodera glycines* Ichinohe) is a devastating pathogen in soybean‐producing regions globally. The economic loss resulting from SCN damage reached US$1.5 billion alone in the United States (Wrather et al., 2001). In China, SCN has been reported in almost every soybean‐producing area and is considered as one of the most important factors restricting soybean yields (Dong et al., 2008; Hua et al., 2018; Lu et al., 2006). Soybean cyst nematode infection suppresses plant growth and causes stunting and chlorosis (Noel, 1999). Infective second‐stage juveniles (J2s) hatch from eggs and seek the host plant depending on signals released from roots or rhizospheric microorganisms; J2s move to root tip, penetrate into the root, and establish feeding sites by injecting nematode secretory compounds into the plant cells. The feeding site, a syncytium, provides nutrition for nematode development, which culminates in the formation of a cyst full of eggs (Mitchum & Baum, 2008; Niblack et al., 2006).

The combination of host plant resistance and crop rotation is an effective way to control SCN, whereas few resistance varieties and limited land resources restrict nematode management. In the United States, 90% resistance sources are derived from PI 88788 (Patil et al., 2019). In northeastern China, the main resistance sources are from Peking (Hua et al., 2018; Tian et al., 2019b). The presence of multiple SCN races or HG types in the field makes SCN control more difficult. In China, SCN race 3 has been considered as a major race in northeastern China, the largest soybean production area, and races 1 and 4 are the major races in the Huanghuai‐Hai region, the other major soybean production area (Li et al., 2011). More than 10 SCN races and 10 HG Types have been reported in China (Hua et al., 2018; Wang, 2019). However, virulence shift (e.g. races 4, 14) of SCN populations has been reported and resistance loss was found in northeastern China (Chen et al., 2015; Hua et al., 2018; Tian et al., 2007; Yang et al., 2015). The major resistant cultivars, Kangxian series, originally bred for SCN race 3 resistance, does not have resistance to the newly identified races 4 and 14 in northeastern China (Hua et al., 2018). A new and super‐virulent race (X12) was found to be able to reproduce on all indicator lines for SCN race and HG Type identification in Shanxi Province in China (Lian et al., 2017). Similarly, SCN virulence shift resulting in resistance losses resulting from continuous planting of single‐source resistance in the same field is also common in the United States (Acharya et al., 2016; Hershman et al., 2008; Mitchum et al., 2007; Tylka & Mullaney, 2015; Vuong et al., 2010).

Soybean cyst nematode resistance in soybean is a multigenic and quantitative trait (Concibido et al., 2004). A total of 213 quantitative trait loci (QTL) have been identified through biparental QTL related by ontology and 125 QTL obtained through genome‐wide association study (GWAS) of soybean accessions that cover all 20 chromosomes (http://www.soybase.org). Among them, two major QTL were mapped to chromosomes (Chrs) 18 (*rhg1*) and 8 (*Rhg4*), respectively, which contribute to SCN resistance in various soybean cultivars; the copy number variations in the region of *rhg1* (31 kb) or *Rhg4* (35.7 kb) play roles in SCN resistance or susceptibility (Cook et al., 2012, 2014; Liu et al., 2012, 2017; Patil et al., 2019). One of the genes, *Glyma.18G022500*, at the *rhg1* locus encodes an α‐soluble N‐ethylmaleimide‐sensitive factor attachment protein (SNAP) called *GmSNAP*18, in which single nucleoid polymorphisms (SNPs) are able to differentiate resistance or susceptibility (Liu et al., 2017). The Peking‐type *rhg1‐a* has lower copy numbers (2–4), PI88788‐type *rhg1‐b* has higher copy numbers (>6), and susceptible ‘William82’ has one copy (Brucker et al., 2005; Cook et al., 2012, 2014; Kadam et al., 2016; Patil et al., 2019). The higher copy numbers in *Rhg4* leads to a broad‐based resistance (Patil et al., 2019). The gene *Glyma.08G108900* (called *GmSHMT08*) encoding a serine hydroxymethyl transferase is responsible for *Rhg4* resistance (Liu et al., 2012). The promoter regions of the two genes, *GmSNAP18* and *GmSHMT08*, are also involved in SCN resistance or susceptibility (Patil et al., 2019). Besides the two genes, numerous other QTL for SCN resistance denote the complexity of the trait. The combination of other genes with the *rhg1* and *Rhg4* genes were also reported to confer resistance to different races. Tian et al. (2019a) identified that a codominant CAPS marker, GmSNAP11‐2565 detected in the gene *Glyma.11g234500* (JGI: 32,967,943–32,972,705 bp in reference genome W82a2.v2) combined with *GmSNAP18* (*rhg1*) and *GmSHMT08* (*Rhg4*) provided high resistance to SCN race 3. Suzuki et al. (2020) also mapped one locus, *rhg2* on Chr 11 (expanded 32.2–33.0 Mbp in W82 genome) flanked by two markers, Sat_123 and BARCSOYSSR11_1420, which contributed high resistance to SCN race 1 when combined with the *rhg1* and *Rhg4* resistance derived from PI 84751 in Japan. Since the two QTL from the two studies fall into the same region, they might be same QTL, which can combine with the two major genes (*rhg1* and *Rhg4*) to produce the highest resistance compared with each gene alone. So many reported GWAS‐identified QTL from soybean accessions signify the natural resistance characteristics from different genetic backgrounds. The extent of resistance depends on different combinations of major or minor genes in various genetic backgrounds. However, less is known about how the interactions of these minor genes play a role in nematode resistance in the absence of the major resistance genes, such as *rhg1* and *Rhg4*.

Chromosome segment substitution lines (CSSLs) are developed by backcrossing (BC) to a recurrent parent, which is usually an adapted or popular genotype, with a relative distant or wild donor genotype carrying valuable genes following genome‐wide marker‐assisted selection and then selfing. Each line represents a genetic stock for a specific segment of chromosome from the donor and possesses a uniform genetic background as its recurrent parent. The advanced generations of CSSLs (above BC_3_) resemble recombinant inbred lines (RILs), thus representing a permanent population. The CSSLs are able to enhance genetic diversity and to provide ideal resources for crop improvement, QTL mapping, detection of epistatic interactions, gene identification and discovery as well (Balakrishnan et al., 2019). Importantly, the phenotypic change with specific segments in CSSLs compared with the recurrent parent can reveal the sequence and position of candidate genes corresponding with the trait. Therefore, minor or major QTL can be identified efficiently through phenotypic screening CSSLs. Approximately 75 CSSLs in 17 major crops have been reported such as rice (*Oryza sativa* L.), wheat (*Triticum aestivum* L.), maize (*Zea mays* L.), pearl millet [*Cenchrus americanus* (L.) Morrone], barley (*Hordeum vulgare* L.), soybean, tomato (*Solanum lycopersicum* L.), upland cotton (*Gossypium hirsutum* L.) and others (Balakrishnan et al., 2019). Two soybean CSSL populations advanced to BC_5_ or BC_6_ derived from soybean × wild soybean (donor) from China were available (Jiang et al., 2020; Wang et al., 2013; Xin et al., 2016; Yang et al., 2017) and one BC_3_F_2_ CSSL population from a cross between Japanese soybean cultivar Enrei as a recurrent parent and Chinese soybean Peking as a donor was reported (Watanabe et al., 2018). Of these, one population containing 195 BC_3_–BC_5_F_2_ CSSLs was derived from a recurrent parent NN1138‐2 (*G. max*) which adapt to southern China, and a wild soybean N24852 (donor, *G. soja*). Quantitative trait loci or segments were detected for contribution to seed traits including length, width, roundness, perimeter, cross‐section area (Yang et al., 2017), and plant height (Zhang et al., 2018). The second CSSL population from the cross between a popular cultivar Suinong 14 (100‐grain weight, 21 g) in Heilongjiang Province, northeastern China and one wild‐type donor parent ZYD00006 (100‐grain weight, 2.8 g), was developed for more than 10 yr to BC_6_F_2_ in 2014; the total length of substituted segments in Suinong14 was ∼1,865.17 cM and the coverage rate of substituted segments was 82.43% (Jiang et al., 2020). The CSSLs were used for QTL mapping of protein (Ma et al., 2014; Yin et al., 2016), seed traits (Chen et al., 2014; Mao et al., 2014; Wei et al., 2016; Xin et al., 2016; Zeng et al., 2012), nodulation (Wang et al., 2020b), drought, and low temperature (Zhang et al., 2012) but not for nematode resistance. The yield‐related traits (plant height, 100‐seed weight, seed weight per plant) of introgression lines displayed transgressive characters beyond the recurrent parent Suinong14 phenotypes (Jiang et al., 2020). Compared with numerous reports for QTL mapping of yield or other morphological traits (e.g. plant height, flower, seed size, and shape) using CSSLs, QTL associated with biotic stress traits have received less attention such as in rice (Wang et al., 2020c; Zhu et al., 2014), maize (Lopez‐Zuniga et al., 2019; Martins et al., 2019), and cotton (Ulloa et al., 2016). Transgressive progenies compared with the recurrent parental line were also observed in these biotic stress traits. Recombinant inbred lines, backcrossing, CSSLs, or F_2_ populations derived from both susceptible parents were reported to produce transgressive resistance to root‐knot nematode (RKN, *Meloidogyne incognita*) in cotton (Ulloa et al., 2016; Wang et al., 2012, 2017). Our previous screening displayed that both Suinong14 and ZYD00006 were susceptible to HG Types 1.3, 2.5.7, and 1.2.3.5.6.7 (Hua et al., 2018). The question here was whether these CSSLs with the introgression of wild‐type genome could suppress nematode production compared with the recurrent parent Suinong14. If so, what and how many QTL are involved in SCN resistance trait and where are these QTL localized on the chromosomes?

**Core Ideas**

- CSSLs are suitable for QTL mapping *Heterodera glycines* resistance trait in soybean.
- Transgressive resistance was found in CSSLs.
- For the first time, QTL associated with cysts per gram root have been detected.
- Positive alleles for female index and cysts per gram root exhibited low nematode reproduction.

Further, since more than 300 QTL including GWAS QTL were reported for SCN resistance covering all 20 chromosomes, implying multiple minor effect QTL, these chromosome segment substitution lines should aid in identifying new QTL and confirming the reported QTL associated with nematode resistance. The high‐throughput genotyping by whole‐genome resequencing, a robustly genetic screening method for SNP discovery, genome profiling, and QTL mapping, will provide accurate and high‐resolution physical maps for facilitating QTL fine mapping in CSSLs (He et al., 2014; Jarquín et al., 2014; Zhang et al., 2019).

Currently, soybean response to SCN is evaluated with female index (FI) determined in greenhouse or field assays where the average number of female cysts on a tested genotype is divided by the average number of female cysts on a susceptible control ‘Lee’ or designed susceptible genotypes (Niblack et al., 2002). Female index is based on the number of cysts in each plant but not associated with root weight. Generally speaking, root weight varies among genotypes, especially small roots for wild‐type accessions. It is well known that the evaluation of RKN or reniform nematode production in plants usually uses egg masses per gram root, eggs per gram root, or females per gram root (Li et al., 2018; Stetina & Young, 2006; Wang et al., 2006). However, it is less certain whether FI is always consistent with cysts per gram root (CGR), if they are controlled by same or different QTL, and what the correlation is between FI and CGR. A large difference of root weight between the two parental lines provided the opportunity to study the effects of CGR among CSSLs.

Hence, the aims of this study were to use the CSSLs (Suinong14 × ZYD00006) for QTL mapping of SCN FI and CGR through genome resequencing and phenotyping; to determine QTL correlation between FI and CGR; to confirm the reported QTL and discover new QTL for marker‐assisted selection, and to identify nematode resistant or tolerant soybean lines for breeding programs.

## 2 MATERIALS AND METHODS

### 2.1 Plant materials

Two hundred and thirteen CSSLs including mixed backcross populations of BC_3_F_7_, BC_4_F_6_, BC_5_F_5_, BC_6_F_4_, and BC_7_F_3_ were used for whole‐genome resequencing. Of these, 162 lines with enough seeds were screened for nematode resistance. As mentioned above, these CSSLs had been confirmed with the introgression of chromosome segments from wild‐type ZYD00006 (donor parent) to the cultivar Suinong14 (recurrent parent) background based on marker‐assisted selection (Jiang et al., 2020; Xin et al., 2016). Soybean Lee 68 was used as a susceptible control for nematode resistance evaluation and Dongsheng1 as the nematode culture host.

### 2.2 Nematode culture and phenotypic evaluation in CSSLs

Soybean cyst nematode HG type 2.5.7 (race 5) was cultured on susceptible cultivar Dongsheng1 for inoculation and the inoculation procedure followed the method by Hua et al. (2018). The cysts were collected from roots and soil at 35 d after inoculation; eggs were released by crushing cysts; then eggs were obtained through 800–150 μm nested sieves, and J2s were hatched from eggs on a six to eight layers of tissue paper supported by a screen placed on a petri dish or a hatching dish containing sterile water with 3 mM ZnSO_4_ for stimulating hatching at 28 ℃. The hatched J2s moved through the tissue paper into the hatching dish. After 3–4 d, J2s were collected and counted for nematode inoculation. The response of CSSLs to the nematode were evaluated at 22–28 ℃, photoperiod of 16 vs. 8 h, day vs. night, under controlled greenhouse conditions run by automatic computer system in Northeast Institute of Geography and Agroecology, Chinese Academy of Sciences. Two seeds were sown in an 8‐cm‐diam., 12‐cm‐height plastic pot containing sterilized soil and sand (1:1 ratio) and thinned to one seedling at 3–4 d after planting. Eight‐day seedlings was inoculated with 1,000 J2s in 1 ml suspension. Each line was composed of six replications and plants were arranged in a randomized block design. Because J2s were inoculated instead of eggs, the plants were harvested at 21 d and roots were gently rinsed and weighted. The cysts on each plant were counted under light by naked eyes. The number of CGR for each plant was calculated with the number of cysts divided by fresh root weight. Female index was used for evaluation of the SCN resistance scale and calculated as follows: FI = (mean value of females on testing line/mean value of females on susceptible control Lee 68) × 100. The SCN resistance scale was determined by the approach of Schmitt and Shannon (1992): FI ≤ 10 (resistant), FI = 11–30 (moderately resistant), FI = 31–60 (moderately susceptible), and FI > 60 (susceptible). The phenotypic evaluation of CSSLs was carried out in September 2018 and repeated in April 2020.

### 2.3 High‐throughput genotyping by whole‐genome resequencing

Genomic DNA from the leaf tissue of 213 CSSLs and two parents was extracted by the CTAB (cetyl trimethylammonium bromide) method (Peng et al., 2015). At least 1 μg of DNA from each sample was used for whole‐genome resequencing with Illumina conducted by Biomarker Technologies (Beijing, China). A 30× coverage sequence for parents and a 5× coverage sequence reads were generated for each CSSL. The reads were aligned to the reference genome of William82 (*G. max Wm82.a2.v1*, http://www.soybase.org) via the program Burrows–Wheeler Aligner (Li & Durbin, 2009). The alignments were transformed to the sequence alignment map format and assigned into a binary alignment map files using a Samtools‐based pipeline (Li et al., 2009). Detection of SNPs was performed with the Genome Analysis Toolkits (GATK) software (https://www.broadinstitute.org/gatk/guide/). A slide‐window approach was performed for SNP variant calling errors and to calculate the ratio of SNP alleles derived from ZYD00006 and Suinong14 (Huang et al., 2009). A window size of 17 SNPs and a step size of one SNP were used to scan the genotypic data. Windows with more than 12 SNPs from ZYD00006 (aa) and windows with more than 14 SNPs from Suinong14 (bb) were considered to be homozygous, and others were considered heterozygous (ab). A single block was designed when adjacent windows contained the same genotypes, and if with different genotypes at adjacent windows, a recombinant break point was recognized. A segment of consecutive SNPs without a recombination event represented one haplotype block (bin marker) which can be served as one genetic marker. The starting points of physical positions of bin markers were changed to mega base pairs (Mbp) format and then all bin markers on each chromosome were formed into a bin map for the following QTL detection.

### 2.4 QTL detection

Quantitative trait loci associated with root weight, CGR, and FI were detected by MapQTL 5.0 (Van Ooijen, 2004). Single‐marker analysis was performed by applying nonparametric mapping (Kruskal–Wallis analysis [*K* ^*^]) test, which was equal to the one‐way analysis of variance. The significant (major) QTL were set up as *P* < .005. If the major QTL was also detected in the other test at least at *P* < .05 with *K* ^*^ test, it was considered as one common QTL in both tests. The major QTL identified at least in one of the two tests were listed out. Mapping, based on multiple‐QTL models (MQM), was also conducted to detect phenotypic variance explained (PVE) of root weight, CGR, and FI for each haplotype block. Logarithm of odds (LOD) scores ≥2 were listed out for each detected block corresponding with *K* ^*^ test for QTL associated with FI and CGR. The threshold of LOD scores for root weight were determined after 1,000 permutation tests for each chromosome at α = 0.05 (Churchill & Doerge, 1994). Once a major QTL associated with FI or CGR in either test was determined in a chromosome, all other major or minor QTL with at least *P* < .1 in either test were chosen in that chromosome. In other words, all haplotype blocks (bin markers) linked to major or minor QTL in that chromosome were kept for map construction with MapChart 2.3 (Voorrips, 2002) and unlinked blocks were discarded. For the naming of QTL, *Hg5‐Gs*/*GmXXa*/*b‐FI*/*CGR* was designated as each QTL; *Hg5* represents *H. glycines* race 5, *Gs* for positive allele from *G. soja*, *Gm* for positive allele from *G. max*, XX for chromosome number, followed with labels a or b if there are two or more QTL in the same chromosome, FI for female index, and CGR for cysts per gram root.

### 2.5 Statistical analyses

Analysis of data from phenotyping screening was conducted by one‐way analysis of variance (ANOVA) by applying SAS 9.4 (SAS Institute Inc.). Comparison among cyst number and FI of different treatments was performed by Tukey's HSD test (*P* < .05) and data obtained were used for QTL analyses. Correlation among FI, CGR, and root weight was constructed with linear trendline in Microsoft Excel 2013.

## 3 RESULTS

### 3.1 Phenotypic response of three soybean genotypes and CSSLs to *Heterodera glycines*

Significant difference (*P* < .05) was found in CGR and FI among the two parents (Suinong14 and ZYD00006) and the susceptible control Lee 68. Wild‐type ZYD00006 displayed greater numbers of CGR (267.26 ± SE 10.57) than Suinong14 (137.57 ± SE 17.42) and the susceptible control Lee 68 (123.23 ± SE 19.21) but showed lower FI (68.56 ± SE 10.58) than Suinong14 (112.93 ± SE 7.29) and Lee 68 (100 ± SE 11.73). There was no significance in CGR and FI between Suinong14 and Lee 68.

**FIGURE 1:**
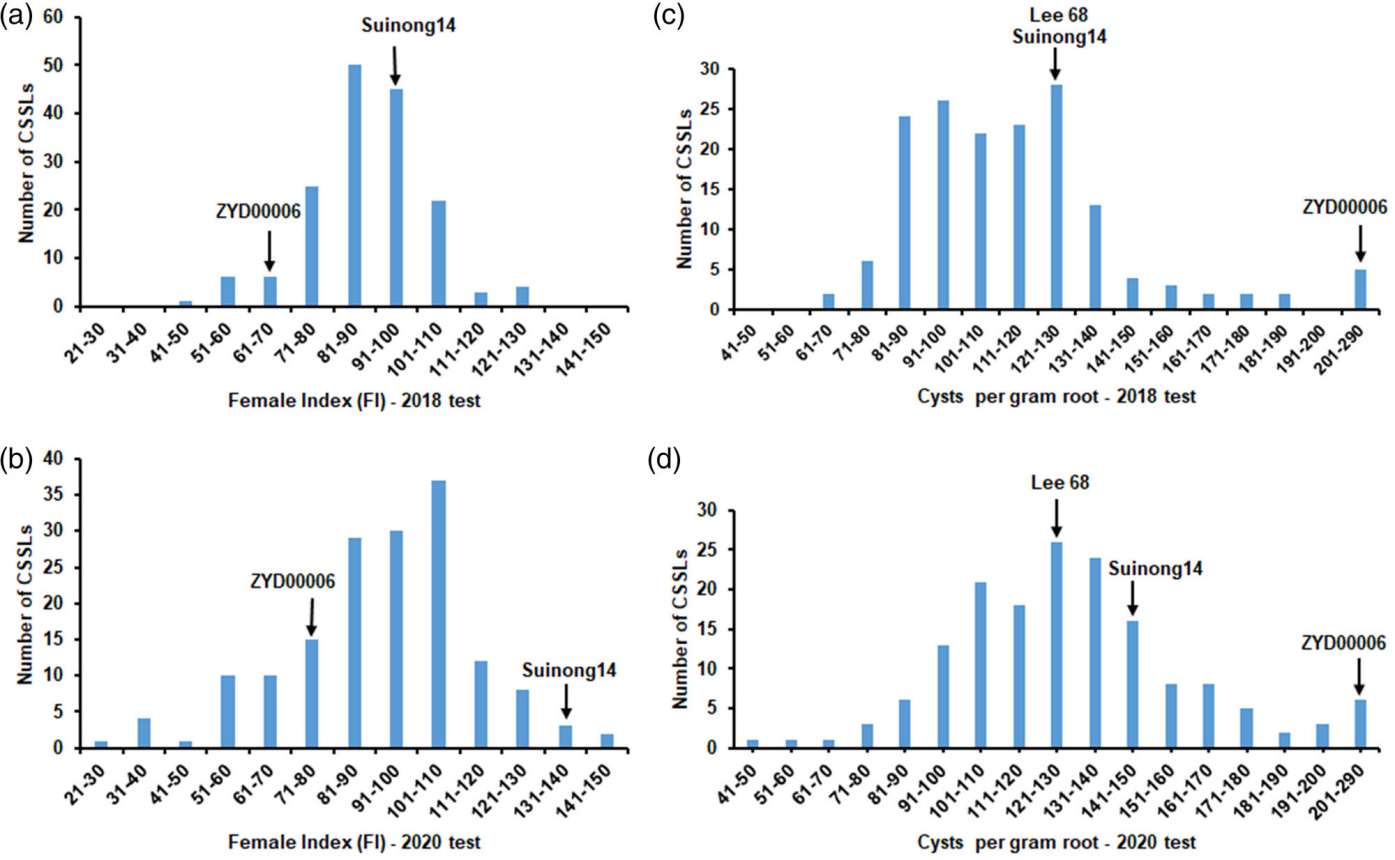
Distributions of *Heterodera glycines* (HG) female index (FI) and cysts per gram root (CGR) for soybean cyst nematode HG Type 2.5.7 (race 5) in chromosome segment substitution populations (CSSLs) derived from the recurrent parent *Glycine max* Suinong14 and the wild donor parent *G. soja* ZYD00006

The phenotypic response of 162 CSSLs to HG Type 2.5.7 in both tests (2018 and 2020) is shown in Figure 1a, b for FI and Figure 1c, d for CGR. The two tests had similar wide‐range distributions of phenotypic response (Figure 1, Supplemental Table S1). The mean CGR among all tested lines from the two tests was 122.95 (range from 68.76–255.23) and the mean number of FI was 90.30 (range from 42.58–121.62), denoting that the introgression of chromosome segments from the wild‐type genome to the local cultivar Suinong14 resulted in phenotypic change. The CSSLs had a maximum 62.3 and 50% decrease in FI (CSSL 55) and CGR (CSSL 189), respectively, and an 85.5% increase in CGR (CSSL 124) when compared with Suinong14.

One hundred thirty‐two CSSLs had fewer CGRs than Suinong14 (CGR, 137.57) and three lines (55, 89, and 189) with FI ≤ 60 exhibited moderate susceptibility, lower than both ZYD00006 (FI, 68.56) and Suinong14 (FI, 112.93), indicating transgressive inheritance.

### 3.2 Construction of haplotype blocks and QTL mapping of FI and CGR

**FIGURE 2:**
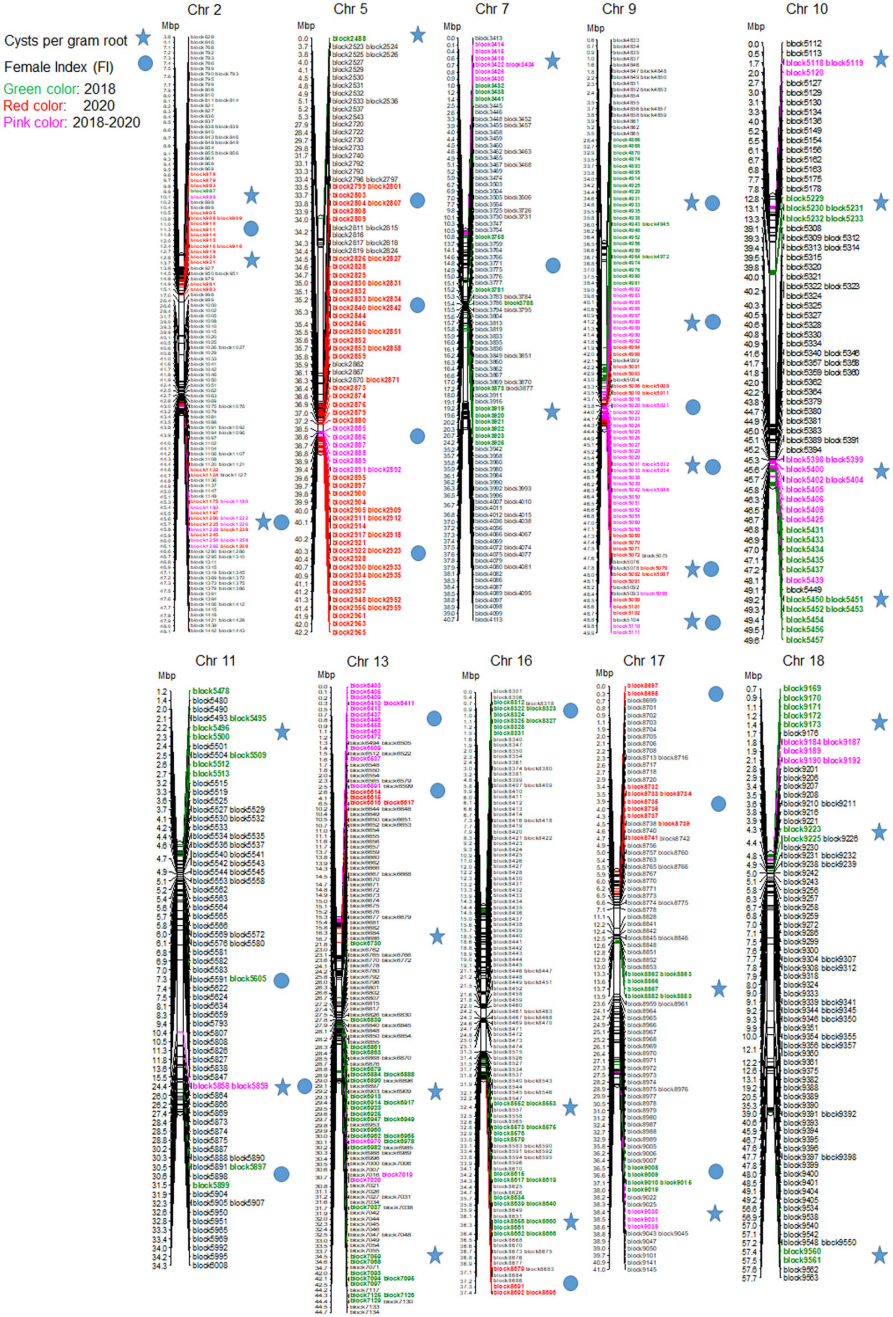
The positions of major quantitative trait loci (QTL) associated with *Heterodera glycines* female index (FI) and cysts per gram root (CGR) located on each chromosome haplotype blocks for chromosome segment substitution populations (CSSLs) between the recurrent parent Suinong14 and the donor parent ZYD00006. All haplotype blocks displayed here had at least *P* < .1 (Kruskal–Wallis analysis, *K* ^*^ test) for either FI or cysts per gram root trait. The significant (major) QTL were set as *P* < .005 in either 2018 or 2020 test

In total, 3,780 haplotype blocks containing 580,524 SNPs were identified on 20 chromosomes with an average of 189 blocks (ranged from 117 to 259) on each chromosome through genome resequencing of 213 CSSLs and two parental lines. The 162 CSSLs tested in this study contained 3,766 blocks distributed on 20 chromosomes. A total of 33 significant QTL in either test with *P* < .005 were detected on 18 chromosomes to contribute resistance to HG Type 2.5.7 FI and CGR by *K* ^*^ test (Table 1, Supplemental Table S2). Four QTL (*Hg5‐Gs05‐FI*, *Hg5‐Gs09‐FI*, *Hg5‐Gs11‐FI*, and *Hg5‐Gs13‐FI*) associated with FI were revealed on Chrs 5, 9, 11, and 13 and eight QTL (*Hg5‐Gm01‐CGR*, *Hg5‐Gs02a‐CGR*, *Hg5‐Gm07‐CGR*, *Hg5‐Gm09‐CGR*, *Hg5‐Gm10‐CGR*, *Hg5‐Gm11‐CGR*, *Hg5‐Gs13‐CGR*, and *Hg5‐Gm18‐CGR*) linked with CGR were detected on Chrs 1, 2, 7, 9, 10, 11, 13, and 18 in both the 2018 and 2020 tests (Table 1). Out of all identified QTL, those on Chr 9 (*Hg5‐Gs09‐FI* and *Hg5‐Gm09‐CGR*) and Chr 11 (*Hg5‐Gs11‐FI* and *Hg5‐Gm11‐CGR*) contributed nematode suppression to both FI and CGR, but the introgression of the ZYD00006 alleles to Suinong14 background had reverse functions because FI was lower and CGR was higher in CSSLs when compared with those trait values in Suinong14 (Table 1). The positions of major QTL (*P* < .005) associated with FI and CGR are displayed in the reference physical maps with haplotype blocks containing polymorphic SNPs between the two parental lines (Figure 2). Twenty‐five out of 33 QTL mapped by *K* ^*^ test displayed LOD score ≥2 when using MQM mapping analysis (Table 1). One QTL associated with FI on Chr 7b and four QTL associated with CGR on Chrs 3, 5, 6, and 14 were found in MQM mapping with LOD ≥ 2 but not significantly (*P* < .005) detected by *K* ^*^ test (Table 1). Of them, seven QTL accounted for 5.6–14.2% PVE, and eight QTL accounted for 5.9–15.5% PVE for FI in the 2018 and 2020 tests, respectively, and 14 QTL contributed to 6.1–36.2% PVE, and nine QTL contributed to 6.1–16.1% PVE for CGR in the 2018 and 2020 tests, respectively (Table 1, Supplemental Table S3). A total of 38 QTL by both *K* ^*^ test and MQM mapping were identified on 20 chromosomes (Table 1).

**TABLE 1:** Quantitative trait loci (QTL) associated by nonparametric mapping (Kruskal–Wallis analysis, *K* ^*^) and multiple‐QTL model (MQM) mapping with *Heterodera glycine* (Hg) female index (FI) and cysts per gram root (CGR) in chromosome segment substitution populations (CSSLs) derived from the backcrossing of *Glycine max* cv. Suinong14 (SN) with the donor parent *G. soja* ZYD00006 (ZYD) in 2018 and 2020 tests

				2018 *K* ^*^ test			2020 *K* ^*^ test			2018 MQM			2020 MQM		
QTL name^a^	Chr^b^	QTL region^c^	SNP block	*K* ^*^	ZYD^e^	SN	*K* ^*^	ZYD	SN	LOD^f^	PVE	Additive effect	LOD	PVE	Additive effect
		MBP									%			%	
QTL‐FI															
* Hg5‐Gs05‐FI*	5	33.5–42.2	2799–2965	5.02**	82.2	90.2	16.81****** ^*^	71.2	95.4	–	–	–	5.9	15.5	−12.02
* Hg5‐Gs09‐FI*	9	28.4–50.2	4866–5111	10.12****	74.7	90.3	19.85****** ^*^	69.8	95.0	3.9	10.5	–7.81	5.8	15.2	−12.63
* Hg5‐Gs011‐FI*	11	24.4–25.4	5858–5859	14.67******	65.8	90.0	4.41**	73.8	92.7	5.4	14.2	–12.13	–	–	–
* Hg5‐Gs013‐FI*	13	0.01–10.1	6403–6617	6.38**	81.0	91.0	9.15****	86.1	96.9	–	–	–	2.2	5.9	−5.39
* Hg5‐Gs02‐FI*	2	8.8–15.2	843–983	5.15**	86.0	90.5	11.42*****	66.1	93.6	–	–	–	3.3	9.0	−13.70
* Hg5‐Gs04‐FI*	4	51.2–52.0	2475–2486	5.35**	81.4	90.0	11.31*****	74.7	94.3	–	–	–	3.4	9.1	−9.78
* Hg5‐Gs15‐FI*	15	50.7–51.8	8280–8300	–	–	–	7.98****	70.0	92.7	–	–	–	–	–	–
* Hg5‐Gs16a‐FI*	16a	37.1–37.9	8679–8696	–	–	–	13.23******	63.7	92.8	–	–	–	3.1	8.5	−15.64
* Hg5‐Gs17‐FI*	17	0–4.7	8697–8741	–	–	–	9.75****	79.0	94.7	–	–	–	2.9	7.8	−8.02
* Hg5‐Gs01‐FI*	1	2.2–4.2	278–343	10.48****	80.0	91.0	–	–	–	3.7	9.9	–5.53	–	–	–
* Hg5‐Gs07a‐FI*	7a	10.8–15.5	3758–3788	8.30****	75.5	89.3	–	–	–	2.0	5.6	–7.06	–	–	–
* Hg5‐Gs12‐FI*	12	32.3–38.6	6275–6347	10.01****	76.9	89.8	–	–	–	2.5	7.0	–6.18	–	–	–
* Hg5‐Gs16b‐FI*	16b	0.7–1.5	8312–8331	12.01*****	72.5	90.3	–	–	–	4.5	11.9	–8.88	–	–	–
* Hg5‐Gm17‐FI*	17	36.5–37.1	9008–9010	7.93****	98.7	87.5	–	–	–	2.4	6.6	5.80	–	–	–
* Hg5‐Gs07b‐FI*	7b	0.67–2.5	3422–3441	–	–	–	5.94**	72.05	93.4	–	–	–	2.4	6.7	−11.5
QTL‐CGR															
* Hg5‐Gm01‐CGR*	1	0.01–0.8	1–64	10.20****	146.8	111.3	4.40**	148.4	127.6	4.4	11.8	17.57	–	–	–
* Hg5‐Gs02a‐CGR*	2a	8.8–13.7	854–921	8.27****	96.9	118.2	11.28*****	94.9	132.7	–	–	–	2.6	7	−19.95
* Hg5‐Gm07‐CGR*	7	0.15–2.5	3414–3441	15.28****** ^*^	192.2	110.6	4.20**	156.5	127.9	13.4	31.6	45.47	–	–	–
* Hg5‐Gm09‐CGR*	9	32.7–50.2	4868–5111	15.55****** ^*^	188.3	110.7	5.44**	164.6	128.0	12.7	30.2	38.31	2.4	6.7	18.02
* Hg5‐Gm10‐CGR*	10	45.3–48.3	5398–5439	17.65****** ^*^	191.1	110.0	5.29**	165.2	127.7	15.8	36.2	40.53	3.1	8.4	19.30
* Hg5‐Gm11‐CGR*	11	24.4–25.4	5858–5859	11.55*****	206.7	111.5	7.89****	175.4	128.3	13.9	32.7	47.64	2.9	8.0	23.58
* Hg5‐Gs13‐CGR*	13	27.8–44.4	6839–7129	20.45****** ^*^	86.2	118.6	7.80***	73.6	131.6	2.6	7.1	–15.36	–	–	–
* Hg5‐Gm18‐CGR*	18	1.8–2.2	9184–9192	9.41****	172.2	112.7	14.10******	182.1	126.0	4.7	12.4	21.32	6.2	16.1	24.27
* Hg5‐Gs02b‐CGR*	2b	45.3–46.1	1175–1266	–	–	–	11.45*****	95.7	132.4	–	–	–	2.2	6.1	−18.31
* Hg5‐Gs04‐CGR*	4	10.2–10.5	2225–2226	8.40****	99.4	118.2	–	–	–	–	–	–	–	–	–
* Hg5‐Gs08‐CGR*	8	40.7–47.7	4707–4832	13.14******	97.5	118.2	–	–	–	–	–	–	–	–	–
* Hg5‐Gs14‐CGR*	14	2.1–7.5	7226–7351	12.50******	91.6	117.8	–	–	–	–	–	–	–	–	–
* Hg5‐Gs15‐CGR*	15	17.4–33.0	7810–8010	13.86******	97.6	118.5	–	–	–	–	–	–	–	–	–
* Hg5‐Gs16‐CGR*	16	32.4–36.5	8552–8666	16.11****** ^*^	94.5	119.1	–	–	–	2.4	6.6	–12.60	–	–	–
* Hg5‐Gs17‐CGR*	17	13.3–14.0	8862–8883	15.01******	87.0	117.7	–	–	–	–	–	–	–	–	–
* Hg5‐Gm17‐CGR*	17	37.1–38.7	9015–9039	12.64******	141.4	114.3	–	–	–	–	–	–	–	–	–
* Hg5‐Gs19‐CGR*	19	46.7–47.1	10243–10265	9.66****	102.1	120.4	–	–	–	–	–	–	–	–	–
* Hg5‐Gs20‐CGR*	20	37.3–40.0	10959–11077	15.38****** ^*^	92.7	118.3	–	–	–	2.2	6.1	–18.83	–	–	–
* Hg5‐Gm20‐CGR*	20	47.3–47.9	11186	10.85*****	165.0	111.6	–	–	–	6.6	17	26.67	–	–	–
* Hg5‐Gm03‐CGR*	3	3.6–32.7	1526‐1792	5.31**	156.9	110.9	4.37**	156.3	127.3	5.4	14.2	21.22	2.4	6.5	14.37
* Hg5‐Gm05‐CGR*	5	38.5–42.2	2885‐2965	6.913***	149.2	110.9	4.14**	154.1	126.5	5.4	14.1	20.19	2.5	7	13.57
* Hg5‐Gm06‐CGR*	6	46.9–48.6	3350‐3358	5.33**	142.1	112.3	–	–	–	2.8	7.7	12.82	–	–	–
* Hg5‐Gm14‐CGR*	14	44.0–47.6	7543‐7605	7.50***	175.9	113.8	7.67***	151.4	128.2	9.4	23.5	63.94	3.9	10.5	42.75

^a^ QTL–FI: QTL associated with female index phenotype; QTL–CGR, QTL associated with cysts per gram root phenotype. *Hg5‐Gs05‐FI*, the name of QTL for FI on Chr 05 from *Glycine soja* (Gs) ZYD00006 to *Heterodera glycines* race 5 (HG Type 2.5.7). Similarly*, Hg5‐Gm07‐CGR* represents the name of QTL for cysts per gram root on Chr 07 from *G. max* cv. Suinong14 to *H. glycines* race 5.

^b^ Chr, soybean chromosome designation.

^c^ QTL region (Mbp) starts from the physical position of the first detected SNP block to the end of physical position of the last SNP block.

^e^ The values under ZYD and SN represent mean values of phenotype (FI or CGR) associated with ZYD00006 alleles or Suinong14 alleles.

^f^ LOD, logarithm of odds scores and the percentage of phenotypic variance explained (PVE) are based on MQM mapping analysis and the value of LOD and PVE values were chosen in the SNP block with the highest LOD score in the introgression QTL region. The additive effect with positive or negative values means increase or decrease in phenotypic values after the introgression segment of ZYD00006 alleles into Suinong14 background.

** Significant at the .05 probability level.

*** Significant at the .01 probability level.

**** Significant at the .005 probability level.

***** Significant at the .001 probability level.

****** Significant at the .0005 probability level.

### 3.3 QTL mapping of root weight and the association with nematode reproduction

**FIGURE 3:**
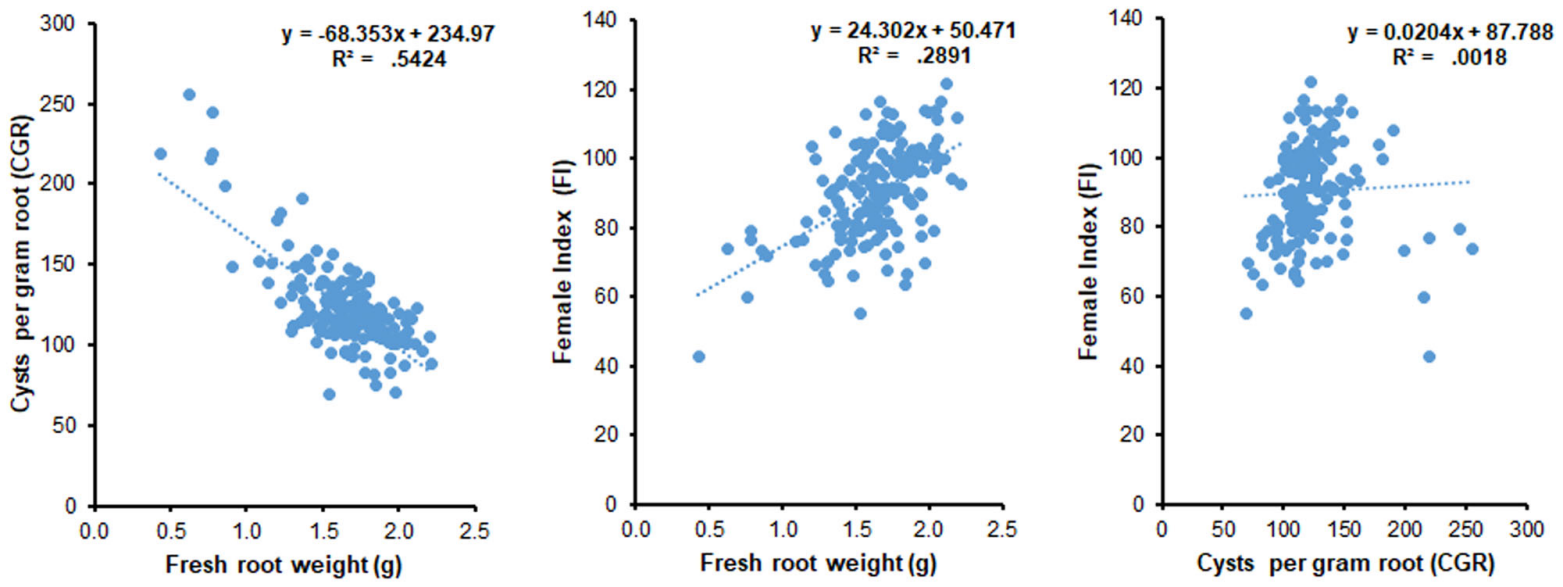
The correlation of *Heterodera glycines* cysts per gram root (CGR), female index (FI), and root weight from the two tests (2018 and 2020, *n* = 12) in 162 chromosome segment substitution populations (CSSLs) derived from the recurrent parent *Glycine max* Suinong14 and the donor parent *G. soja* ZYD00006

ZYD00006 and some CSSLs had lower FI but greater numbers of CGR than Suinong14 because ZYD00006 and some CSSLs had much smaller root systems. The average fresh root weight for CSSLs from the two tests was 1.64 g (range 0.43–2.21 g), while the average root weights of Suinong14 and ZYD00006 were 1.79 ± SE 0.15 and 0.68 ± SE 0.14, respectively. Among CSSLs, unsurprisingly, a higher correlation (*R^2^* = 0.5424) was identified between CGR and root weight than that between FI and root weight (*R^2^* = 0.2891) and that between CGR and FI (*R^2^* = 0.0018) (Figure 3). To understand whether the CGR trait was linked with the root weight trait, MQM mapping analysis was conducted for the average root weight from the two tests. Since the permutation test determined the threshold of LOD score between 2.0 and 3.1 on each chromosome, QTL with the LOD score ≥2 were listed in Table 3 and Supplemental Table S4. A total of 29 QTL were identified on 18 chromosomes that contributed 5.5–28.3% PVE to root weight with additive effect. Five out of 29 QTL on Chrs 5, 7, 9, 10, and 11 accounted for >20% PVE for root weight (LOD score 8.5–11.7) and four of them on Chrs 5, 7, 9, and 11 were mapped to the same genomic intervals with both FI and CGR (Table 1). Thirteen other QTL (Table 2) contained the similar genomic regions as those only with CGR on Chrs 1, 3(2), 6, 10, 13, 14 (2), 17 (2), 18, and 20 (2) (Table 1), and three QTL on Chrs 1, 4, and 17 only with FI (Tables 1 and 2).

**TABLE 2:** Quantitative trait loci (QTL) associated by multiple‐QTL model (MQM) mapping with fresh root weight (g) in chromosome segment substitution populations (CSSLs)

Chr^a^	QTL region^b^	SNP block	LOD^c^	ZYD^d^	SN14	PVE^e^	Additive effect^f^
	Mbp			g			
1	0.012–4.220	1–343	3.37	1.40	1.67	9.1	−0.13779
2	3.806–4.190	628–646	3.45	1.56	1.75	9.4	−0.09418
3	4.289–7.071	1555–1597	6.2	1.27	1.68	16.2	−0.20705
3	11.627–32.702	1676–1792	5.99	1.28	1.68	15.7	−0.19819
4	11.314–12.125	2250–2253	3.77	1.37	1.68	10.2	−0.15122
4	51.166–52.030	2475–2486	6.48	1.32	1.69	16.8	−0.18436
5	38.536–42.220	2885–2965	8.54	1.25	1.69	21.5	−0.22113
6	46.872–48.625	3350–3358	2.94	1.44	1.68	8.0	−0.11647
7	0.007–2.499	3413–3441	10.32	1.05	1.68	25.4	−0.31322
8	1.863–3.372	4166–4196	2.66	1.85	1.61	7.3	0.121206
8	15.088–15.647	4484–4500	2.07	1.91	1.61	5.7	0.149401
9	28.384–50.171	4866–5111	9.76	1.25	1.70	24.2	−0.22513
10	0.022–2.410	5112–5120	4.63	1.28	1.67	12.3	−0.19517
10	44.664–48.264	5380–5439	11.72	1.05	1.68	28.3	−0.3167
11	10.213–10.744	5805–5810	2.72	1.40	1.67	7.4	−0.13372
11	24.383–25.442	5858–5859	10.21	0.93	1.67	25.2	−0.37276
13	21.788–34.569	6730–7067	2.69	1.91	1.61	7.4	0.150781
14	5.614–7.715	7297–7363	2.76	1.91	1.61	7.6	0.150683
14	44.424–47.638	7547–7605	5.99	0.73	1.66	15.7	−0.46488
15	20.490–22.324	7839–7867	2.04	1.81	1.61	5.6	0.102394
17	2.678–3.012	8718–8720	2.35	1.37	1.66	6.5	−0.14662
17	13.261–13.469	8862–8863	2.53	1.93	1.62	6.9	0.158043
17	38.755–38.980	9043–9047	2.2	1.26	1.65	6.1	−0.1968
18	0.705–1.245	9169–9171	2.97	1.93	1.61	8.1	0.158188
18	1.801–4.351	9184–9223	2.81	1.31	1.66	7.7	−0.17373
18	9.340–10.019	9350–9354	2.01	1.38	1.66	5.5	−0.13892
19	3.627–5.996	9635–9636	2.45	1.30	1.66	6.7	−0.17854
20	35.908–40.091	10791–11080	2.28	1.86	1.61	6.3	0.123448
20	47.260–47.898	11184–11186	4.97	1.25	1.67	13.2	−0.20917

^a^ Chr, chromosome.

^b^ The QTL region (Mbp) starts from the physical position of the first detected SNP block to the end of physical position of the last SNP block.

^c^ QTL associated with root weight were detected based on LOD (Logarithm of odds ratio) score ≥2 (The threshold of LOD scores with 1000 permutation tests for all chromosomes were between 2–3).

^d^ ZYD, ZYD00006; SN14, Suinong14; indicates the mean value of root weight associated with the ZYD00006 allele or the Suinong14 allele based on the peak LOD score in the corresponding QTL region.

^e^ The percentage of phenotypic variance explained (PVE) is based on the MQM mapping analysis and the peak values of LOD and PVE were chosen in the SNP block in the introgression QTL region.

^f^ The additive effect with positive or negative values indicates increase or decrease in root weight, respectively, after the introgression segment of ZYD00006 allele into Suinong14 background.

To dissect the distribution of QTL with different levels of root weight, FI, and CGR, 13 CSSLs were chosen to identify the substitution of ZYD00006 segments (Table 3). Each of four lines, 55, 124, 89, and 156 with root weight 0.43, 0.63, 0.76, and 0.78 g; CGR 219.20, 255.23, 215.15, and 219.11; FI 42.58, 73.89, 59.63 and 76.73, respectively, demonstrated nine to 13 QTL that negatively affect root weight, five to 11 QTL negatively associated with CGR, and four to seven QTL positively inhibiting FI. Of these, five to 11 QTL negatively associated with both root weight and CGR were mapped to the same chromosome regions, which resulted in small size of roots and great numbers of CGR (Tables 2 and 3). On the contrary, three lines, 189, 214, and 98 with lower CGR but greater root weight than the three lines 124, 89, and 156 carried two to four QTL negatively affecting root weight and two to eight QTL positively associated with CGR (Table 3).

**TABLE 3:** The effect of introgression segments from the donor parent *Glycine soja* ZYD00006 to *G. max* cv. Suinong14 on fresh root weight, cysts per gram root (CGR) and female index (FI) in the individual chromosome segment substitution population (CSSL) plants

**CSSL**	**Traits** **^a^**	**2018**	**2020**	**Mean**	**Chromosome (linkage map)** **^b^**																			
					**1**	**2**	**3**	**4**	**5**	**6**	**7**	**8**	**9**	**10**	**11**	**12**	**13**	**14**	**15**	**16**	**17**	**18**	**19**	**20**
					**D1a**	**D1b**	**N**	**C1**	**A1**	**C2**	**M**	**A2**	**K**	**O**	**B1**	**H**	**F**	**B2**	**E**	**J**	**D2**	**G**	**L**	**I**
189	RW	1.40	1.68	1.54		**–**		**–**	**–**				**–**											
	CGR	92.0	45.5	68.76		**+**			**–**			**+**	**–**					**+**		**+**				
	FI	75.1	34.8	54.97		**+**			**+**				**+**				**+**			**+**	**+**			
98	RW	1.60	2.08	1.84	**–**			**–**						**–**			**+**		**+**			**+**		**+**
	CGR	79.4	84.6	81.99				**+**				**+**					**+**	**+**	**+**	**+**			**+**	**+**
	FI	68.9	57.7	63.30	**+**			**+**	**+**										**+**	**+**				
214	RW	1.75	1.96	1.85			**–**	**–**				**+**					**+**							
	CGR	83.9	66.4	75.13			**–**	**+**									**+**							
	FI	85.1	48.1	66.58													**+**	**+**						
180	RW	1.29	2.37	1.83	**–**	**–**									**–**		**+**							**+**
	CGR	108.3	112.0	110.16	**–**																		**+**	**+**
	FI	83.6	107.2	95.38	**+**												**+**				**+**			
218	RW	1.57	2.29	1.93		**–**																		
	CGR	93.4	114.8	104.11																**+**				
	FI	86.5	105.9	96.20																**+**				
154	RW	1.12	1.46	1.29		**–**			**–**						**–**		**+**							
	CGR	123.7	94.0	108.9		**+**			**–**							**+**	**+**							
	FI	76.5	56.9	66.72		**+**			**+**								**+**				**+**			
168	RW	1.47	1.53	1.50		**–**																		
	CGR	157.9	120.8	139.37		**+**																		
	FI	125.4	82.9	104.15		**+**																		
73	RW	1.59	1.14	1.36		**–**			**–**						**–**		**+**	**–**						
	CGR	111.4	270.5	190.93					**–**								**+**	**–**				**–**		
	FI	99.9	115.7	107.81					**+**								**+**							
115	RW	1.23	1.18	1.21		**–**	**–**	**–**	**–**		**–**		**–**	**–**	**–**			**–**			**–**			
	CGR	168.2	187.3	177.75			**–**		**–**		**–**		**–**	**–**				**–**	**+**		**–**		**+**	
	FI	121.0	86.0	103.47					**+**				**+**						**+**		**–**			
156	RW	0.72	0.85	0.78		**–**	**–**	**–**		**–**	**–**		**–**	**–**							**–**		**–**	
	CGR	220.6	217.7	219.11			**–**	**+**			**–**		**–**	**–**							**–**			
	FI	79.8	73.7	76.73				**–**			**+**		**+**			**+**	**+**		**+**	**+**	**–**			
89	RW	0.56	0.96	0.76	**–**	**–**	**–**	**–**	**–**	**–**	**–**		**–**	**–**	**–**									**–**
	CGR	206.3	224.0	215.15	**–**		**–**	**+**	**–**	**–**	**–**		**–**	**–**	**–**							**–**		**–**
	FI	62.4	56.9	59.63	**+**				**+**				**+**		**+**		**+**							
124	RW	0.61	0.65	0.63	**–**	**–**	**–**	**–**	**–**				**–**	**–**	**–**			**–**				**–**		
	CGR	282.0	228.5	255.23	**–**		**–**	**+**	**–**				**–**	**–**	**–**			**–**				**–**		
	FI	88.7	59.1	73.89					**+**				**+**		**+**		**+**							
55	RW	0.36	0.49	0.43	**–**	**–**	**–**	**–**	**–**	**–**	**–**		**–**	**–**	**–**			**–**				**–**		**–**
	CGR	268.1	170.3	219.20	**–**		**–**	**+**	**–**	**–**	**–**		**–**	**–**	**–**			**–**				**–**		**–**
	FI	49.0	36.2	42.58	**+**			**+**	**+**				**+**		**+**		**+**			**+**				

^a^ RW, Root weight (g); CGR, Cysts per gram root; FI: female index. The values of fresh root weight (g), FI, and cysts/gram root in each line are the average values from the two tests (2018 and 2020).

^b^ The symbols + and – for root weight indicate positive allele from ZYD00006 to increase root weight and negative allele to decrease root weight based on the QTL identified in Table 2, respectively. On the contrary, the symbols + and – for CGR and FI represents ZYD00006 allele positively or negatively inhibit nematode reproduction based on the QTL identified in Table 1, respectively.

## 4 DISCUSSION

This study represents the first time for QTL mapping of CGR and the first to use CSSLs to evaluate FI and CGR for *H. glycines*. Based on the resistance classification with FI (Schmitt & Shannon, 1992), the two parental lines were susceptible to HG Type 2.5.7. A wide range of FI and CGR were distributed in CSSLs (Figure 1), and a maximum of 62.3 and 50% decrease in FI and CGR, respectively, and an 85.5% increase in CGR were found in CSSLs when compared with the recurrent parent Suinong14, indicating that the chromosome segment substitution of Suinong14 with wild‐type ZYD00006 changed the SCN phenotype. Quantitative trait loci mapping revealed a total of 38 QTL by *K* ^*^ test and MQM mapping with strong additive effect that covered 20 chromosomes from both parents and contributed 5.6–15.5 and 6.1–36.2% PVE to SCN FI and CGR resistance from the two tests, respectively (Table 1).

Through the comparison of previously published QTL based on the physical positions (http://www.soybase.org), 33 out of 38 QTL (Table 1) were reported and five QTL are unique (Table 4). Among the 33 QTL, a total of 107 published QTL were found with a range from 1 to 40 QTL for each mapped QTL position in CSSLs. Interestingly, the QTL *Hg5‐Gm18‐CGR* was mapped on Chr 18, where the *rhg1* localizes and 40 QTL associated with response to SCN were reported in the *rhg1* region (http://www.soybase.org). The ZYD00006 allele substitution in this QTL interval region on Chr 18 to Suinong14 resulted in an increase of ∼50% for the number of CGR in both tests (Table 1). These results confirmed CSSLs are excellent population type to detect and validate QTL controlling complex traits (Balakrishnan et al., 2019; Nadeau et al., 2000; Zhang et al., 2019; Zhou et al., 2017; Zhu et al., 2017). The introduction of donor genome segments to the recurrent parent not only results in novel phenotypic variation but also reduces genetic background noise, which is especially helpful in finding QTL with additive minor effects as in this study since these small additive effects can be masked by QTL with large effects in traditional populations such as F_2_ and RIL segregating populations (Ali et al., 2010; Balakrishnan et al., 2020; Ebitani et al., 2005; Keurentjes et al., 2007; Yamamoto et al., 2009; Zhu et al., 2020). Ulloa et al. (2016) screened cotton CSSLs to validate segment substitution regions from *Gossypium barbadense* L. to *G. hirsutum* background, which contributed to RKN resistance and *Fusarium* wilt resistance. Therefore, CSSLs can be considered as valuable resources for breeding programs and powerful genetic analysis tools to determine QTL effects for resistance.

**TABLE 4:** The reported quantitative trait loci (QTL) associated with *Heterodera glycines* (Hg) female index (FI) in the mapped QTL regions contributed resistance to FI and cysts per gram root (CGR)

QTL name	Reported QTL (soybase.org)
QTL–FI	
* Hg5‐Gs05‐FI*	SCN 18‐1, SCN 5‐g10
* Hg5‐Gs09‐FI*	SCN 39‐5, SCN 34‐3, SCN 1‐g6, SCN 5‐g20, SCN 6‐g6
* Hg5‐Gs11‐FI*	SCN 2‐1, SCN 23‐1, SCN 24‐1, SCN 26‐1
* Hg5‐Gs13‐FI*	SCN 5‐g28
* Hg5‐Gs02‐FI*	SCN 4‐g2, SCN 5‐g3, SCN 6‐g1
* Hg5‐Gs04‐FI*	no
* Hg5‐Gs15‐FI*	SCN 25‐1
* Hg5‐Gs16a‐FI*	SCN 44‐1, SCN 44‐9, SCN 44‐13
* Hg5‐Gs17a‐FI*	SCN 23‐2
* Hg5‐Gs01‐FI*	SCN 19‐3, SCN 20‐3, SCN 21‐2
* Hg5‐Gs07a‐FI*	SCN 1‐g3, SCN 5‐g36
* Hg5‐Gs12‐FI*	SCN 4‐g9
* Hg5‐Gs16b‐FI*	SCN 6‐g7
* Hg5‐Gm17b‐FI*	SCN 5‐g42
* Hg5‐Gs07b‐FI*	SCN5‐g16
QTL–CGR	
* Hg5‐Gm01‐CGR*	SCN 19‐3, SCN 20‐3, SCN 21‐2
* Hg5‐Gs02a‐CGR*	SCN 4‐g2, SCN 5‐g3, SCN 6‐g1
* Hg5‐Gm07‐CGR*	SCN5‐g16
* Hg5‐Gm09‐CGR*	SCN 34‐3, SCN 39‐5, SCN 1‐g6, SCN 5‐g20, SCN 6‐g6
* Hg5‐Gm10‐CGR*	SCN 4‐g7, SCN 1‐g9, SCN 5‐g21
* Hg5‐Gm11‐CGR*	SCN 2‐1, SCN 23‐1, SCN 24‐1, SCN 26‐1
* Hg5‐Gs13‐CGR*	SCN 1‐g12, SCN 1‐g13, SCN 4‐g11
* Hg5‐Gs16‐CGR*	SCN 1‐2, SCN 5‐2, SCN 28‐2, SCN 28‐4, SCN 29‐2, SCN 29‐6, SCN 38‐3, SCN 38‐7, SCN 44‐1, SCN 44‐9, SCN 44‐13, SCN 5‐g38
* Hg5‐Gm18‐CGR*	SCN 4‐4, SCN 5‐1, SCN 6‐2, SCN 13‐1, SCN 14‐2, SCN 23‐3, SCN 24‐2, SCN 25‐2, SCN 26‐3, SCN 28‐1, SCN 28‐3, SCN29‐3, SCN 29‐4, SCN 29‐5, SCN29‐8, SCN 30‐1, SCN 30‐2, SCN 32‐1, SCN 33‐1, SCN 33‐3, SCN 33‐5, SCN 36‐3, SCN 36‐4, SCN 36‐5, SCN 38‐2, SCN 39‐3, SCN 39‐7, SCN 40‐2, SCN 41‐1, SCN 41‐2, SCN 41‐3, SCN 44‐3, SCN 44‐16, SCN 45‐1, SCN 47‐1, SCN 48‐2, SCN 48‐3, SCN 50‐4, SCN 1‐g19, SCN 1‐g20
* Hg5‐Gs02b‐CGR*	no
* Hg5‐Gs04‐CGR*	no
* Hg5‐Gs08‐CGR*	SCN 37‐4, SCN 50‐2, SCN 5‐g18
* Hg5‐Gs14‐CGR*	SCN 4‐g12, SCN 3‐g11, SCN 17‐2, SCN 19‐2
* Hg5‐Gs15‐CGR*	SCN 25‐1, SCN 5‐g34
* Hg5‐Gm17a‐CGR*	no
* Hg5‐Gm17b‐CGR*	no
* Hg5‐Gs19‐CGR*	SCN 29‐7
* Hg5‐Gs20‐CGR*	SCN 34‐2
* Hg5‐Gm20‐CGR*	SCN 44‐8, SCN 44‐12
* Hg5‐Gm03‐CGR*	SCN 5‐g7, SCN 4‐3, SCN 44‐4, SCN 44‐15
* Hg5‐Gm05‐CGR*	SCN 5‐g10
* Hg5‐Gm06‐CGR*	SCN 5‐g14, SCN 17‐3, SCN 20‐2, SCN 42‐3
* Hg5‐Gm14‐CGR*	SCN 10‐1

Unsurprisingly, no QTL linked to the two major resistance gene loci *rhg1* or *Rhg4* in the CSSLs were identified to contribute to FI resistance since both Suinong14 and ZYD00006 display susceptible response to SCN FI. However, the segment substitution in the *rhg1* region on Chr 18 increased CGR by 50% compared with Suinong14 (Table 1), suggesting the *rhg1* region in Suinong14 had a minor effect on CGR and adding an unfavorable allele from ZYD00006 leads to more nematode reproduction. Similarly, five more QTL on Chrs 5, 7, 9, 10, and 11 negatively affected CGR with the substitution of ZYD00006 allele in both tests, denoting unfavorable introgression alleles. Coincidentally, QTL on Chrs 5, 7, 9, 10, and 11 also negatively contributed >20% PVE to root weight with the introgression of ZYD00006 allele, suggesting unfavorable loci linked together to control root weight and CGR. The dissection of individual CSSLs, for example, lines 55, 89, 115, 124, and 156 (Table 3), confirmed unfavorable linkage loci for both root weight and CGR. These lines exhibited lower FI, but the smaller roots and greater CGR compared with Suinong14 make the CSSLs not ideal genotypic stocks for a breeding program. On the contrary, the individual CSSLs carrying more positive alleles from ZYD00006 associated with three traits (root weight, FI, and CGR) will be potential breeding resources for crop improvement compared with Suinong14, such as the CSS lines 98, 189, and 214 (Table 3). The CSS line 214 carrying only two positive ZYD00006 alleles for each trait of root weight, CGR and FI (Table 1), displayed lower CGR and FI, implying epistatic effect. These results indicate that breeding genotypes with both low CGR and FI will be an ideal choice in the absence of strong resistance genes *rhg1* and *Rhg4*. Thus, factors affecting both root weight and cyst number per plant should be considered for phenotypic evaluation in the presence of wide range of variation of root weight and in the lack of major effect genes (e.g. *rhg1* and *Rhg4*). Success has been achieved by evaluating RKN reproduction with eggs or egg masses per gram plant root (Atamian et al., 2012), for example, in pepper (*Capsicum annuum* L. var. *annuum*) (Kiewnick et al., 2009), tomato (*Solanum lycopersicum* L.) (Huang et al., 2004), cotton (Wang et al., 2006), rice (Galeng‐Lawilao et al., 2018), potato (*Solanum tuberosum* L.) (Mao et al., 2019), soybean (Li et al., 2018), and others. Further, the different combinations of these minor QTL produced wide variation in phenotype among CSSLs, which directly confirms the presence of multiple minor QTL in the soybean accessions through GWAS or biparental population analysis, even in the susceptible genotype and also indirectly explains the wide range of phenotypic variation among soybean accessions carrying the same major resistance genes *rhg1* and *Rhg4* (Patil et al., 2019). However, the classification of CGR into different categories (R, MR, MS and S) will require more research with a wide range of phenotypes, including root weight, cysts per plant, or CGR.

The CSSLs in the Suinong14 background have been tested for protein, seed size, plant height, yield, nodulation, drought and low temperature traits as mentioned above and transgressive segregation in lines was also observed for each trait. Therefore, the combined knowledge from these traits will facilitate choosing lines with preferred traits to improve soybean yield and quality. Most main cultivars are susceptible and the limited number of resistant varieties (e.g. Kangxian series) are only planted in certain accumulated temperature zones in Heilongjiang Province (Hua et al., 2018). The identified CSSLs with low FI and CGR might be potential candidates for replacement of susceptible varieties when resistance varieties are not available. The identified lines with extreme phenotypes can also be chosen for further gene identification and functional analysis of candidate genes associated with the traits. In soybean, Zou et al. (2020) used two identified CSSLs derived from the recurrent parent Suinong14 as described in this study that exhibited contrasting phenotypes for response to bacterial blight (*Xanthomonas vasicola*) to find that two candidate genes in the CSSLs play a vital role in response to bacterial blight. In cucumber (*Cucumis sativus* L.), one CSSL was found to carry powdery mildew resistance genes, and whole‐genome resequencing of this line with the two parental lines revealed large numbers of SNPs and insertion–deletions; further gene expression analysis determined candidate genes associated with resistance to powdery mildew (Xu et al., 2016; Xu et al., 2017). Here, the significant SNP blocks contained predicted candidate genes that can be studied functionally to determine the association with SCN resistance.

Gene interactions from two parents that produce phenotypes in the progeny beyond of the parent phenotype range is called transgressive segregation (Rieseberg et al., 2003). Transgressive inheritance is an important way to obtain novel or enhanced levels of pathogen resistance for crop improvement. Numerous traits with transgressive inheritance have been reported (Bell & Travis, 2005; Rieseberg et al., 2003). In this study, the CSSLs carrying better performance for lower FI and CGR than Suinong14 and ZYD00006 are typical transgressive segregants. Transgressive resistance had been observed in soybean–SCN system (Dias et al., 2005) and soybean–brown stem rot (Perez et al., 2010) in which highly resistant progenies were identified from the cross between resistant and susceptible parents. However, no reports are available for studies between two susceptible parents. One well‐studied case for transgressive resistance is cotton–RKN system. Transgressive segregation is very common in cotton, and recombination between resistant and susceptible or between susceptible and susceptible parents in intraspecific crosses *G. hirsutum* × *G. hirsutum* or interspecific cross *G. barbadense* × *G. hirsutum* produces transgressive resistance to RKN but the interspecific cross generates transgressive resistance in earlier generations than the intraspecific cross (Wang et al., 2008; Wang et al., 2012, 2017). Genetic analysis and sequence composition of bacterial artificial chromosome clones in the transgressive RKN resistance and susceptible chromosome regions in cotton revealed resistance gene clusters present in both resistant and susceptible homologous regions but the presence of multiple copies of R proteins, unique insertion–deletion in nucleotide‐binding domain shared by APAF‐1, R proteins and CED‐4 domain, different copies of leucine‐rich repeat domain, and transposable elements in the resistance region might be major factors contributing to complex recombination and transgressive resistance (Wang et al., 2015, 2020a). Forty major and minor QTL were identified for contributing 4.5–12.3% PVE to RKN transgressive resistance in one interspecific RIL cotton population derived from two susceptible parents (Wang et al., 2012). Similarly, 38 QTL contributing 5.6–36.2% PVE to SCN FI and CGR in soybean in this study were detected for SCN resistance, some QTL were identified only in MQM based on multiple‐QTL‐models but no significance (*P < .005*) was found in *K* ^*^ test based on single‐marker analysis and vice versa (Table 1), suggesting transgressive progeny in CSSLs with epistatic effect inhibited nematode production compared with the susceptible parent Suinong14. Further study of small segment substitutions from ZYD00006 will shed more light about epistatic effect genes involved in the SCN‐soybean interaction. These QTL will be used for marker‐assisted selection for SCN resistance breeding programs.

## CONFLICT OF INTEREST

The authors declare that they have no conflict of interest.

## AUTHOR CONTRIBUTIONS

Minghui Huang: Data curation; Formal analysis; Investigation; Methodology; Validation; Visualization; Writing‐original draft. Ruifeng Qin: Data curation; Investigation. Chunjie Li: Data curation; Methodology; Resources. Chunyan Liu: Data curation; Resources. Ye Jiang: Investigation. Jinyao Yu: Investigation. Doudou Chang: Investigation. Philip A. Roberts: Conceptualization; Writing‐review & editing. Qingshan Chen: Conceptualization; Data curation; Funding acquisition; Project administration; Resources; Supervision; Writing‐review & editing. Congli Wang: Conceptualization; Data curation; Formal analysis; Funding acquisition; Project administration; Resources; Supervision; Validation; Writing‐review & editing.

## Supporting information


**Supplemental Table S1** The response of CSSLs to *Heterodera glycines* infection including root fresh weight, cysts per gram root (CGR) and Female Index (FI) at 2018 and 2020 tests.

**Supplemental Table S2** QTL mapping by Kruskal‐Wallis analysis (K^*^ test) of *Heterodera glycine* female index (FI) and cysts per gram root (CGR) in CSSLs derived from *Glycine max* cv. Suinong14 (bb) with *G. soja* ZYD00006 (aa) in 2018‐ and 2020‐tests.

**Supplemental Table S3** MQM mapping of *Heterodera glycine* female index (FI) and cysts per gram root (CGR) in CSSLs derived from *Glycine max* cv. Suinong14 with *G. soja* ZYD00006 in 2018‐ and 2020‐tests

**Supplemental Table S4** MQM mapping of fresh root weight (2018 and 2020 tests) in CSSLs derived from *Glycine max* cv. Suinong14 with *G. soja* ZYD00006.

## References

[tpg220091-bib-0001] Acharya, K. , Tande, C. , & Byamukama, E. (2016). Determination of *Heterodera glycines* virulence phenotypes occurring in South Dakota. Plant Disease, 100, 2281–2286. 10.1094/PDIS-04-16-0572-RE 30682916

[tpg220091-bib-0002] Ali, M. L. , Sanchez, P. L. , Yu, S. B. , Lorieux, M. , & Eizenga, G. C. (2010). Chromosome segment substitution lines: A powerful tool for the introgression of valuable genes from *Oryza* wild species into cultivated rice (*O. sativa*). Rice, 3, 218–234. 10.1007/s12284-010-9058-3

[tpg220091-bib-0003] Atamian, H. S. , Roberts, P. A. , & Kaloshian, I. (2012). High and low throughput screens with root‐knot nematodes *Meloidogyne* spp. Journal of Visualized Experiments, 61, 3629. 10.3791/3629 PMC3402051 22434014

[tpg220091-bib-0004] Balakrishnan, D. , Surapaneni, M. , Mesapogu, S. , & Neelamraju, S. (2019). Development and use of chromosome segment substitution lines as a genetic resource for crop improvement. Theoretical and Applied Genetics, 132, 1–25. 10.1007/s00122-018-3219-y 30483819

[tpg220091-bib-0005] Balakrishnan, D. , Surapaneni, M. , Yadavalli, V. R. , Addanki, K. R. , Mesapogu, S. , Beerelli, K. , & Neelamraju, S. (2020). Detecting CSSLs and yield QTLs with additive, epistatic and QTL×environment interaction effects from *Oryza sativa* × *O. nivara* IRGC81832 cross. Scientific Reports, 10, 7766. 10.1038/s41598-020-64300-0 32385410 PMC7210974

[tpg220091-bib-0006] Bell, M. A. , & Travis, M. P. (2005). Hybridization, transgressive segregation, genetic covariation, and adaptive radiation. Trends in Ecology & Evolution, 20, 358–361. 10.1038/s41598-020-64300-0 16701394

[tpg220091-bib-0007] Brucker, E. , Carlson, S. , Wright, E. , Niblack, T. , & Diers, B. (2005). *Rhg1* alleles from soybean PI 437654 and PI 88788 respond differentially to isolates of *Heterodera glycines* in the greenhouse. Theoretical and Applied Genetics, 111, 44–49. 10.1007/s00122-005-1970-3 15883792

[tpg220091-bib-0008] Chen, Q. , Jiang, H. , Sun, D. , Liu, C. , Xin, D. , Zeng, Q. , Ma, Z. , & Hu, G. (2014). QTL mapping for 100‐seed weight using wild soybean chromosome segment substitution lines. (In Chinese, with English abstract.). Soybean Science, 33, 154–160. 10.11861/j.issn.1000-9841.2014.02.0154

[tpg220091-bib-0009] Chen, J. , Li, X. , Li, Z. , Zhou, C. , Luo, X. , Wang, D. , Duan, Y. , & Chen, L. (2015). Identification of the virulence type of soybean cyst nematode under continuous cropping in Daqing and Anda. (In Chinese, with English abstract.). Soybean Science, 34, 675–678.

[tpg220091-bib-0010] Churchill, G. A. , & Doerge, R. W. (1994). Empirical threshold values for quantitative traits mapping. Genetics, 138, 963–971. 10.1093/genetics/138.3.963 7851788 PMC1206241

[tpg220091-bib-0011] Concibido, V. C. , Diers, B. W. , & Arelli, P. R. (2004). A decade of QTL mapping for cyst nematode resistance in soybean. Crop Science, 44, 1121–1131. 10.2135/cropsci2004.1121

[tpg220091-bib-0012] Cook, D. E. , Bayless, A. M. , Wang, K. , Guo, X. , Song, Q. , Jiang, J. , & Bent, A. F. (2014). Distinct copy number, coding sequence, and locus methylation patterns underlie *Rhg1*‐mediated soybean resistance to soybean cyst nematode. Plant Physiology, 165, 630–647. 10.1104/pp.114.235952 24733883 PMC4044848

[tpg220091-bib-0013] Cook, D. E. , Lee, T. G. , Guo, X. , Melito, S. , Wang, K. , Bayless, A. M. , Wang, J. , Hughes, T. J. , Willis, D. K. , Clemente, T. E. , Diers, B. W. , Jiang, J. , Hudson, M. E. , & Bent, A. F. (2012). Copy number variation of multiple genes at *Rhg1* mediates nematode resistance in soybean. Science, 338, 1206–1209. 10.1126/science.1228746 23065905

[tpg220091-bib-0014] Dias, W. P. , Campos, V. P. , Kiihl, R. A. S. , Arias, C. A. A. , & Toledo, J. F. F. (2005). Genetic control in soybean of resistance to soybean cyst nematode race 4^+^ . Euphytica, 145, 321–329. 10.1007/s10681-005-1944-1

[tpg220091-bib-0015] Dong, I. , Xu, Y. , Li, C. , Pan, F. , Xie, Y. , Han, Y. , Teng, W. , & Li, W. (2008). Cyst density and subspecies identification of soybean cyst nematode in Heilongjiang province. (In Chinese, with English abstract.) Chinese Journal of Oil Crop Sciences, 30, 108–111.

[tpg220091-bib-0016] Ebitani, T. , Takeuchi, Y. , Nonoue, Y. , Yamamoto, T. , Takeuchi, K. , & Yano, M. (2005). Construction and evaluation of chromosome segment substitution lines carrying overlapping chromosome segments of *indica* rice cultivar ‘Kasalath’ in a genetic background of *japonica* elite cultivar ‘Koshihikari’. Breeding Science, 55, 65–73. 10.1270/jsbbs.55.65

[tpg220091-bib-0017] Galeng‐Lawilao, J. , Kumar, A. , & De Waele, D. (2018). QTL mapping for resistance to and tolerance for the rice root‐knot nematode, *Meloidogyne graminicola* . BMC Genetics, 19, 53. 10.1186/s12863-018-0656-1 30081817 PMC6080554

[tpg220091-bib-0018] He, J. , Zhao, X. , Laroche, A. , Lu, Z. X. , Liu, H. , & Li, Z. (2014). Genotyping‐by‐sequencing (GBS), an ultimate marker‐assisted selection (MAS) tool to accelerate plant breeding. Frontiers in Plant Science, 5, 484. 10.3389/fpls.2014.00484 25324846 PMC4179701

[tpg220091-bib-0019] Hershman, D. E. , Heinz, R. D. , & Kennedy, B. S. (2008). Soybean cyst nematode, *Heterodera glycines*, populations adapting to resistant soybean cultivars in Kentucky. Plant Disease, 92, 1475–1475. 10.1094/PDIS-92-10-1475B 30769560

[tpg220091-bib-0020] Hua, C. , Li, C. , Hu, Y. , Mao, Y. , You, J. , Wang, M. , Chen, J. , Tian, Z. , & Wang, C. (2018). Identification of HG types of soybean cyst nematode *Heterodera glycines* and resistance screening on soybean genotypes in Northeast China. Journal of Nematology, 50, 41–50. 10.21307/jofnem-2018-007 30335911 PMC6909372

[tpg220091-bib-0021] Huang, X. , Feng, Q. , Qian, Q. , Zhao, Q. , Wang, L. , Wang, A. , Guan, J. , Fan, D. , Weng, Q. , Huang, T. , Dong, G. , Sang, T. , & Han, B. (2009). High‐throughput genotyping by whole‐genome resequencing. Genome Research, 19, 1068–1076. 10.1101/gr.089516.108 19420380 PMC2694477

[tpg220091-bib-0022] Huang, X. , McGiffen, M. , & Kaloshian, I. (2004). Reproduction of Mi‐virulent *Meloidogyne incognita* isolates on *Lycopersicon* spp. Journal of Nematology, 36, 69–75. 19262789 PMC2620734

[tpg220091-bib-0023] Jarquín, D. , Kocak, K. , Posadas, L. , Hyma, K. , Jedlicka, J. , Graef, G. , & Lorenz, A. (2014). Genotyping by sequencing for genomic prediction in a soybean breeding population. BMC Genomics, 15, 740. 10.1186/1471-2164-15-740 25174348 PMC4176594

[tpg220091-bib-0024] Jiang, H. , Li, C. , Li, R. , Li, Y. , Yin, Y. , Ma, Z. , Zeng, Q. , Zhang, W. , , Liu, C. , & Chen, Q. (2020). Construction of wild soybean backcross introgression lines. (In Chinese, with English abstract.) Chinese Journal of Oil Crop Sciences, 42, 8–16. 10.19802/j.issn.1007-9084.2020014

[tpg220091-bib-0025] Kadam, S. , Vuong, T. D. , Qiu, D. , Meinhardt, C. G. , Song, L. , Deshmukh, R. , Patil, G. , Wan, J. , Valliyodan, B. , Scaboo, A. M. , Shannon, J. G. , & Nguyen, H. T. (2016). Genomic‐assisted phylogenetic analysis and marker development for next generation soybean cyst nematode resistance breeding. Plant Science, 242, 342–350. 10.1016/j.plantsci.2015.08.015 26566850

[tpg220091-bib-0026] Keurentjes, J. J. B. , Bentsink, L. , Alonso‐Blanco, C. , Hanhart, C. J. , Blankestijn‐De Vries, H. , Effgen, S. , Vreugdenhil, D. , & Koornneef, M. (2007). Development of a near‐isogenic line population of *Arabidopsis thaliana* and comparison of mapping power with a recombinant inbred line population. Genetics, 175, 891–905. 10.1534/genetics.106.066423 17179089 PMC1800614

[tpg220091-bib-0027] Kiewnick, S. , Dessimoz, M. , & Franck, L. (2009). Effects of the *Mi‐1* and the *N* root‐knot nematode‐resistance gene on infection and reproduction of *Meloidogyne enterolobii* on tomato and pepper cultivars. Journal of Nematology, 41, 134–139. 22661786 PMC3365310

[tpg220091-bib-0028] Li, H. , & Durbin, R. (2009). Fast and accurate short read alignment with Burrows–Wheeler transform. Bioinformatics, 25, 1754–1760. 10.1093/bioinformatics/btp324 19451168 PMC2705234

[tpg220091-bib-0029] Li, H. , Handsaker, B. , Wysoker, A. , Fennell, T. , Ruan, J. , Homer, N. , Marth, G. , Abecasis, G. , Durbin, R. , & 1000 Genome Project Data Processing Subgroup (2009). The sequence alignment/map format and SAMtools. Bioinformatics, 25, 2078–2079. 10.1093/bioinformatics/btp352 19505943 PMC2723002

[tpg220091-bib-0030] Li, C. , Wang, J. , You, J. , Wang, X. , Liu, B. , Abe, J. , Kong, F. , & Wang, C. (2018). Quantitative trait loci mapping of *Meloidogyne incognita* and *M. hapla* resistance in a recombinant inbred line population of soybean. Nematology, 20, 525–537. 10.1163/15685411-00003157

[tpg220091-bib-0031] Li, Y. H. , Qi, X. T. , Chang, R. , & Qiu, L. J. (2011). Evaluation and utilization of soybean germplasm for resistance to cyst nematode in China. In A. Sudaric (Ed.), Soybean molecular aspects of breeding (pp. 373–396). InTech. http://www.intechopen.com/books/soybean-molecular-aspects-of-breeding/evaluation-and-utilization-of-soybean-germplasm-for-resistance-to-cyst-nematode-in-china

[tpg220091-bib-0032] Lian, Y. , Guo, J. , Li, H. , Wu, Y. , Wei, H. , Wang, J. , Li, J. , & Lu, W. (2017). A new race (X12) of soybean cyst nematode in China. Journal of Nematology, 49, 321–326. 10.21307/jofnem-2017-079 29062156 PMC5644926

[tpg220091-bib-0033] Liu, S. , Kandoth, P. K. , Lakhssassi, N. , Kang, J. , Colantonio, V. , Heinz, R. , Yeckel, G. , Zhou, Z. , Bekal, S. , Dapprich, J. , Rotter, B. , Cianzio, S. , Mitchum, M. G. , & Meksem, K. (2017). The soybean *GmSNAP18* gene underlies two types of resistance to soybean cyst nematode. Nature Communications, 8, 14822. 10.1038/ncomms14822 PMC5378975 28345654

[tpg220091-bib-0034] Liu, S. , Kandoth, P. K. , Warren, S. D. , Yeckel, G. , Heinz, R. , Alden, J. , Yang, C. , Jamai, A. , El‐Mellouki, T. , Juvale, P. S. , Hill, J. , Baum, T. J. , Cianzio, S. , Whitham, S. A. , Korkin, D. , Mitchum, M. G. , & Meksem, K. (2012). A soybean cyst nematode resistance gene points to a new mechanism of plant resistance to pathogens. Nature, 492, 256–260. 10.1038/nature11651 23235880

[tpg220091-bib-0035] Lopez‐Zuniga, L. O. , Wolters, P. , Davis, S. , Weldekidan, T. , Kolkman, J. M. , Nelson, R. , Hooda, K. S. , Rucker, E. , Thomanson, W. , Wisser, R. , & Balint‐Kurti, P. (2019). Using maize chromosome segment substitution line populations for the identification of loci associated with multiple disease resistance. G3: Genes, Genomes, Genetics, 9, 189–201. 10.1534/g3.118.200866 30459178 PMC6325898

[tpg220091-bib-0036] Lu, W. , Gai, J. , & Li, W. (2006). Sampling survey and identification of races of soybean cyst nematode (*Heterodera glycines* Ichinohe) in Huang‐Huai Valleys. (In Chinese, with English abstract.) Agricultural Sciences in China, 39, 306–312.

[tpg220091-bib-0037] Ma, Z. , Sun, D. , Jiang, H. , Fan, D. , Wang, J. , Zeng, Q. , Liu, C. , Li, T. , Hu, G. , & Chen, Q. (2014). Genotyping and QTL mapping of protein content with wild soybean backcross introgressive lines. (In Chinese, with English abstract.) Chinese Journal of Oil Crop Sciences, 36, 316–322. 10.7505/j.issn.1007-9084.2014.03.004

[tpg220091-bib-0038] Mao, Y. , Hu, Y. , Li, C. , Zhang, H. , Yu, D. , Liu, X. , Yang, G. , & Wang, C. (2019). Molecular identification of *Meloidogyne* species isolated from potato in China and evaluation of the response of potato genotypes to these isolates. Nematology, 21, 847–856. 10.1163/15685411-00003259

[tpg220091-bib-0039] Mao, Y. , Jiang, H. , Liu, C. , Xin, D. , Che, J. , Hu, G. , & Chen, Q. (2014). QTL mapping and epistasis analysis of pods per plant and seeds per plant with an advanced backcross population. (In Chinese, with English abstract.) Soybean Science, 33, 467–472. 10.11861/j.issn.1000-9841.2014.04.0467

[tpg220091-bib-0040] Martins, L. B. , Rucker, E. , Thomason, W. , Wisser, R. J. , Holland, J. B. , & Balint‐Kurti, P. (2019). Validation and characterization of maize multiple disease resistance QTL. G3: Genes, Genomes, Genetics, 9, 2905–2912. 10.1534/g3.119.400195 31300480 PMC6723135

[tpg220091-bib-0041] Mitchum, M. G. , Wrather, J. A. , Heinz, R. D. , Shannon, J. G. , & Danekas, G. (2007). Variability in distribution and virulence phenotypes of *Heterodera glycines* in Missouri during 2005. Plant Disease, 91, 1473–1476. 10.1094/PDIS-91-11-1473 30780744

[tpg220091-bib-0042] Mitchum, M. G. , & Baum, T. J. (2008). Genomics of the soybean cyst nematode‐soybean interaction. In G. Stacey (Ed.) Genetics and genomics of soybean (pp. 321–341). Springer. 10.1007/978-0-387-72299-3_17

[tpg220091-bib-0043] Nadeau, J. H. , Singer, J. B. , Matin, A. , & Lander, E. S. (2000). Analysing complex genetic traits with chromosome substitution strains. Nature Genetics, 24, 221–225. 10.1038/73427 10700173

[tpg220091-bib-0044] Niblack, T. L. , Arelli, P. R. , Noel, G. R. , Opperman, C. H. , Orf, J. H. , Schmitt, D. P. , Shannon, J. G. , & Tylka, G. L. (2002). A revised classification scheme for genetically diverse populations of *Heterodera glycines* . Journal of Nematology, 34, 279–288. 19265945 PMC2620582

[tpg220091-bib-0045] Niblack, T. L. , Lambert, K. N. , & Tylka, G. L. (2006). A model plant pathogen from the kingdom animalia: *Heterodera glycines*, the soybean cyst nematode. Annual Review of Phytopathology, 44, 283–303. 10.1146/annurev.phyto.43.040204.140218 16704359

[tpg220091-bib-0046] Noel, G. R. (1999). Soybean cyst nematode. In G. L. Hartman , J. B. Sinclair , & J. C. Rupe (Eds.), Compendium of soybean diseases (4th ed., pp. 52–53). APS Press.

[tpg220091-bib-0047] Patil, G. B. , Lakhssassi, N. , Wan, J. , Song, L.i , Zhou, Z. , Klepadlo, M. , Vuong, T. D. , Stec, A. O. , Kahil, S. S. , Colantonio, V. , Valliyodan, B. , Rice, J. H. , Piya, S. , Hewezi, T. , Stupar, R. M. , Meksem, K. , & Nguyen, H. T. (2019). Whole‐genome re‐sequencing reveals the impact of the interaction of copy number variants of the *rhg1* and *Rhg4* genes on broad‐based resistance to soybean cyst nematode. Plant Biotechnology Journal, 17, 1595–1611. 10.1111/pbi.13086 30688400 PMC6662113

[tpg220091-bib-0048] Peng, J. , Shi, X. , Sun, Y. , Li, D. , Liu, B. , Kong, F. , & Yuan, X. (2015). QTLMiner: QTL database curation by mining tables in literature. Bioinformatics, 31, 1689–1691. 10.1093/bioinformatics/btv016 25586515

[tpg220091-bib-0049] Perez, P. T. , Diers, B. W. , Lundeen, P. , Tabor, G. M. , & Cianzio, S. R. (2010). Genetic analysis of new sources of soybean resistance to brown stem rot. Crop Science, 50, 2431–2439. 10.2135/cropsci2010.03.0159

[tpg220091-bib-0050] Rieseberg, L. H. , Widmer, A. , Arntz, A. M. , & Burke, B. (2003). The genetic architecture necessary for transgressive segregation is common in both natural and domesticated populations. Philosophical Transactions of the Royal Society B‐Biological Sciences, 358, 1141–1147. 10.1098/rstb.2003.1283 PMC1693210 12831480

[tpg220091-bib-0051] Schmitt, D. P. , & Shannon, G. (1992). Differentiating soybean responses to *Heterodera Glycines* races. Crop Science, 32, 275–277. 10.2135/cropsci1992.0011183X003200010056x

[tpg220091-bib-0052] Stetina, S. R. , & Young, L. D. (2006). Comparisons of female and egg assays to identify *Rotylenchulus reniformis* resistance in cotton. Journal of Nematology, 38, 326–332. 19259536 PMC2586702

[tpg220091-bib-0053] Suzuki, C. , Taguchi‐Shiobara, F. , Ikeda, C. , Iwahashi, M. , Matsui, T. , Yamashita, Y. , & Ogura, R. (2020). Mapping soybean *rhg2* locus, which confers resistance to soybean cyst nematode race 1 in combination with *rhg1* and *Rhg4* derived from PI 84751. Breeding Science, 70, 474–480. 10.1270/jsbbs.20035 32968350 PMC7495202

[tpg220091-bib-0054] Tian, Y. , Liu, B. , Shi, X. , Reif, J. C. , Guan, R. , Li, Y. , & Qiu, L. (2019a). Deep genotyping of the gene *GmSNAP* facilitates pyramiding resistance to cyst nematode in soybean. The Crop Journal, 7, 677–684. 10.1016/j.cj.2019.04.003

[tpg220091-bib-0055] Tian, Z. , Gao, G. , Zhou, C. , Du, Z. , Wu, Y. , Wang, M. , Yang, L. , & Li, J. (2007). Study on the variation of soybean cyst nematode. (In Chinese, with English abstract.) Soybean Science, 26, 291–293. 10.3969/j.issn.1000-9841.2007.02.037

[tpg220091-bib-0056] Tian, Z. , Li, J. , Wu, Y. , Zhou, C. , Yang, L. , Chen, J. , Li, X. , Yu, D. , Ma, L. , Wang, M. , & Yang, L. (2019b). Breeding of a new nematode‐resistant soybean variety Nongqing soybean 20. (In Chinese.) Soybean Science & Technology, 1, 42–44. 10.3969/j.issn.1674-3547.2019.01.012

[tpg220091-bib-0057] Tylka, G. L. , & Mullaney, M. P. (2015). Soybean cyst nematode‐resistant soybean varieties for Iowa (Vol. 1). Extension and Outreach Publications, Iowa State University of Science and Technology. https://store.extension.iastate.edu/product/5154

[tpg220091-bib-0058] Ulloa, M. , Wang, C. , Saha, S. , Hutmacher, R. B. , Stelly, D. M. , Jenkins, J. N. , Burke, J. , & Roberts, P. A. (2016). Analysis of root‐knot nematode and Fusarium wilt disease resistance in cotton (*Gossypium* spp.) using chromosome substitution lines from two alien species. Genetica, 144, 167–179. 10.1007/s10709-016-9887-0 26882892

[tpg220091-bib-0059] Van Ooijen, J. W. (2004). MapQTL®5, Software for the mapping of quantitative trait loci in experimental populations. Kyazma B. V.

[tpg220091-bib-0060] Voorrips, R. E. (2002). MapChart: Software for the graphical presentation of linkage maps and QTLs. Journal of Heredity, 93, 77–78. 10.1093/jhered/93.1.77 12011185

[tpg220091-bib-0061] Vuong, T. D. , Sleper, D. A. , Shannon, J. G. , & Nguyen, H. T. (2010). Novel quantitative trait loci for broad‐based resistance to soybean cyst nematode (*Heterodera glycines* Ichinohe) in soybean PI 567516C. Theoretical and Applied Genetics, 121, 1253–1266. 10.1007/s00122-010-1385-7 20559815

[tpg220091-bib-0062] Wang, C. (2019). Research advances in genetic markers for resistance to soybean cyst nematodes (SCN). Plant Diseases and Pests, 10, 10–14. 10.19579/j.cnki.plant-d.p.2019.01.003

[tpg220091-bib-0063] Wang, C. , Ulloa, M. , Duong, T. T. , & Roberts, P. A. (2017). QTL analysis of transgressive nematode resistance in tetraploid cotton reveals complex interactions in chromosome 11 regions. Frontiers in Plant Science, 8, 1979. 10.3389/fpls.2017.01979 29209344 PMC5702019

[tpg220091-bib-0064] Wang, C. , Ulloa, M. , & Roberts, P. A. (2006). Identification and mapping of microsatellite markers linked to a root‐knot nematode resistance gene (*rkn1*) in Acala NemX cotton (*Gossypium hirsutum* L.). Theoretical and Applied Genetics, 112, 770–777. 10.1007/s00122-005-0183-0 16362274

[tpg220091-bib-0065] Wang, C. , Ulloa, M. , Mullens, T. R. , Yu, J. Z. , & Roberts, P. A. (2012). QTL analysis for transgressive resistance to root‐knot nematode in interspecific cotton (*Gossypium* spp.) progeny derived from susceptible parents. PLoS ONE, 7, e34874. 10.1371/journal.pone.0034874 22514682 PMC3325951

[tpg220091-bib-0066] Wang, C. , Ulloa, M. , Nichols, R. L. , & Roberts, P. A. (2020a). Sequence composition of bacterial chromosome clones in a transgressive root‐knot nematode resistance chromosome region in tetraploid cotton. Frontiers in Plant Science, 11, 574486. 10.3389/fpls.2020.574486 33381129 PMC7767830

[tpg220091-bib-0067] Wang, C. , Ulloa, M. , & Roberts, P. A. (2008). A transgressive segregation factor (*RKN2*) in *Gossypium barbadense* for nematode resistance clusters with gene *rkn1* in *G. hirsutum* . Molecular Genetics and Genomics, 279, 41–52. 10.1007/s00438-007-0292-3 17940800

[tpg220091-bib-0068] Wang, C. , Ulloa, M. , Shi, X. , Yuan, X. , Saski, C. , Yu, J. Z. , & Roberts, P. A. (2015). Sequence composition of BAC clones and SSR markers mapped to upland cotton chromosomes 11 and 21 targeting resistance to soil‐borne pathogens. Frontiers in Plant Science, 6, 791. 10.1007/s10681-012-0817-7 26483808 PMC4591483

[tpg220091-bib-0069] Wang, J. , Wang, J. , Ma, C. , Zhou, Z. , Yang, D. , Zheng, J. , Wang, Q. , Li, H. , Zhou, H. , Sun, Z. , Liu, H. , Li, J. , Chen, L. , Kang, Q. , Qi, Z. , Jiang, H. , Zhu, R. , Wu, X. , Liu, C. , Chen, Q. , & Xin, D. (2020b). QTL mapping and data mining to identify genes associated with the *Sinorhizobium fredii* HH103 T3SS effector NopD in soybean. Frontiers in Plant Science, 11, 453. 10.3389/fpls.2020.00453 32508850 PMC7249737

[tpg220091-bib-0070] Wang, S. , Xie, X. , Zhang, Z. , Guan, H. , Mao, D. , Wu, W. , & Chen, Z. (2020c). Fine mapping of *qBlsr3d*, a quantitative trait locus conferring resistance to bacterial leaf streak in rice. Crop Science, 60, 1854–1862. 10.1002/csc2.20155

[tpg220091-bib-0071] Wang, W. , He, Q. , Yang, H. , Xiang, S. , Zhao, T. , & Gai, J. (2013). Development of a chromosome segment substitution line population with wild soybean (*Glycine soja* Sieb. Et Zucc.) as donor parent. Euphytica, 189, 293–307. 10.1007/s10681-012-0817-7

[tpg220091-bib-0072] Watanabe, S. , Shimizu, T. , Machita, K. , Tsubokura, Y. , Xia, Z. , Yamada, T. , Hajika, M. , Ishimoto, M. , Katayose, Y. , Harada, K. , & Kaga, A. (2018). Development of a high‐density linkage map and chromosome segment substitution lines for Japanese soybean cultivar Enrei. DNA Research, 25, 123–136. 10.1093/dnares/dsx043 29186379 PMC5909467

[tpg220091-bib-0073] Wei, S. , Chen, Q. , Jiang, H. , Yin, Y. , Wang, D. , Hu, G. , Wu, X. , & Pan, X. (2016). QTL mapping for seed weight per plant by using the wild soybean chromosome segment substitution lines. (In Chinese, with English abstract.) Soybean Science, 35, 742–747.

[tpg220091-bib-0074] Wilson, R. F. (2008). Soybean: Market driven research needs. In G. Stacey (Ed.), Genetics and genomics of soybean. Plant genetics and genomics: Crops and models, (Vol. 2, pp. 3–15). Springer.

[tpg220091-bib-0075] Wrather, J. , Stienstra, W. , & Koenning, S. (2001). Soybean disease loss estimates for the United States from 1996 to 1998. Canadian Journal of Plant Pathology, 23, 122–131. 10.1080/07060660109506919

[tpg220091-bib-0076] Xin, D. , Qi, Z. , Jiang, H. , Hu, Z. , Zhu, R. , Hu, J. , Han, H. , Hu, G. , Liu, C. , & Chen, Q. (2016). QTL location and epistatic effect analysis of 100‐seed weight using wild soybean (*Glycine soja* Sieb. & Zucc.) chromosome segment substitution lines. PLoS ONE, 11, e0149380. 10.1371/journal.pone.0149380 26934088 PMC4774989

[tpg220091-bib-0077] Xu, Q. , Shi, Y. , Yu, T. , Xu, X. , Yan, Y. , Qi, X. , & Chen, X. (2016). Whole‐genome resequencing of a cucumber chromosome segment substitution line and its recurrent parent to identify candidate genes governing powdery mildew resistance. PLoS ONE, 11, e0164469. 10.1371/journal.pone.0164469 27764118 PMC5072683

[tpg220091-bib-0078] Xu, Q. , Xu, X. , Shi, Y. , Qi, X. , & Chen, X. (2017). Elucidation of the molecular responses of a cucumber segment substitution line carrying *Pm5.1* and its recurrent parent triggered by powdery mildew by comparative transcriptome profiling. BMC Genomics, 18, 21. 10.1186/s12864-016-3438-z 28056792 PMC5217421

[tpg220091-bib-0079] Yamamoto, T. , Yonemaru, J. , & Yano, M. (2009). Towards the understanding of complex traits in rice: Substantially or superficially? DNA Research, 16, 141–154. 10.1093/dnares/dsp006 19359285 PMC2695773

[tpg220091-bib-0080] Yang, H. , Wang, W. , He, Q. , Xiang, S. , Tian, D. , Zhao, T. , & Gai, J. (2017). Chromosome segment detection for seed size and shape traits using an improved population of wild soybean chromosome segment substitution lines. Physiology and Molecular Biology of Plants, 23, 877–889. 10.1007/s12298-017-0468-1 29158636 PMC5671450

[tpg220091-bib-0081] Yang, L. , Tian, Z. , Zhou, C. , Li, J. , Wu, Y. , Shi, C. , & Zhu, C. (2015). Identification of subspecies of soybean cyst nematode in Daqing and Anda areas. (In Chinese, with English abstract.) Journal of Northeast Agricultural Sciences, 40, 76–79. 10.16423/j.cnki.1003-8701.2015.06.020

[tpg220091-bib-0082] Yin, Y. , Pan, X. , Jiang, H. , Wei, S. , Liu, C. , Hu, G. , & Chen, Q. (2016). Epistatic analysis for protein content using wild soybean backcross introgressive lines. (In Chinese, with English abstract.) Soybean Science, 35, 353–359. 10.11861/j.issn.1000-9841.2016.03.0353

[tpg220091-bib-0083] Zeng, G. , Jiang, H. , Liu, C. , Fan, D. , Ma, Z. , Wang, H. , Hu, G. , & Chen, Q. (2012). Genotype analysis and QTL mapping small seed size soybean with advanced backcross population. (In Chinese, with English abstract.) Chinese Journal of Oil Crop Science, 34, 473–477.

[tpg220091-bib-0084] Zhang, B. , Shang, L. , Ruan, B. , Zhang, A. , Yang, S. , Jiang, H. , Liu, C. , Hong, K. , Lin, H. , Gao, Z. , Hu, J. , Zeng, D. , Guo, L. , & Qian, Q. (2019). Development of three sets of high‐throughput genotyped rice chromosome segment substitution lines and QTL mapping for eleven traits. Rice, 12, 33. 10.1186/s12284-019-0293-y 31076960 PMC6510774

[tpg220091-bib-0085] Zhang, W. B. , Qiu, P. C. , Jiang, H. W. , Liu, C. Y. , Xin, D. W. , Li, C. D. , Hu, G. H. , & Chen, Q. S. (2012). Dissection of genetic overlap of drought and low‐temperature tolerance QTLs at the germination stage using backcross introgression lines in soybean. Molecular Biology Reports, 39, 6087–6094. 10.1007/s11033-011-1423-9 22207180

[tpg220091-bib-0086] Zhang, X. , Wang, W. , Guo, N. , Zhang, Y. , Bu, Y. , Zhao, J. , & Xing, H. (2018). Combining QTL‐seq and linkage mapping to fine map a wild soybean allele characteristic of greater plant height. BMC Genomics, 19, 226. 10.1186/s12864-018-4582-4 29587637 PMC5870336

[tpg220091-bib-0087] Zhou, Y. , Xie, Y. , Cai, J. , Liu, C. , Zhu, H. , Jiang, R. , Zhong, Y. , Zhang, G. , Tan, B. , Liu, G. , Fu, X. , Liu, Z. , Wang, S. , Zhang, G. , & Zeng, R. (2017). Substitution mapping of QTLs controlling seed dormancy using single segment substitution lines derived from multiple cultivated rice donors in seven cropping seasons. Theoretical and Applied Genetics, 130, 1191–1205. 10.1007/s00122-017-2881-9 28283703

[tpg220091-bib-0088] Zhu, D. , Li, X. , Wang, Z. , You, C. , Nie, X. , Sun, J. , Zhang, X. , Zhang, D. , & Lin, Z. (2020). Genetic dissection of an allotetraploid interspecific CSSLs guides interspecific genetics and breeding in cotton. BMC Genomics, 21, 431. 10.1186/s12864-020-06800-x 32586283 PMC7318736

[tpg220091-bib-0089] Zou, J. , Zhang, Z. , Yu, S. , Kang, Q. , Shi, Y. , Wang, J. , Zhu, R. , Ma, C. , Chen, L. , Wang, J. , Li, J. , Li, Q. , Liu, X. , Zhu, J. , Wu, X. , Hu, Z. , Qi, Z. , Liu, C. , Chen, Q. , & Xin, D. (2020). Responses of soybean genes in the substituted segments of segment substitution lines following a *Xanthomonas* infection. Frontiers in Plant Science, 11, 972. 10.3389/fpls.2020.00972 32719700 PMC7351525

[tpg220091-bib-0090] Zhu, S. , Huang, R. , Wai, H. P. , Xiong, H. , Shen, X. , He, H. , & Yan, S. (2017). Mapping quantitative trait loci for heat tolerance at the booting stage using chromosomal segment substitution lines in rice. Physiology and Molecular Biology of Plants, 23, 817–825. 10.1007/s12298-017-0465-4 29158631 PMC5671447

[tpg220091-bib-0091] Zhu, Y. , Zuo, S. , Chen, Z. , Chen, X. , Li, G. , Zhang, Y. , Zhang, G. , & Pan, X. (2014). Identification of two major rice sheath blight resistance QTLs, *qSB1‐1HJX74* and *qSB11HJX74*, in field trials using chromosome segment substitution lines. Plant Disease, 98, 1112–1121. 10.1094/PDIS-10-13-1095-RE 30708790

